# TAZ2 truncation confers overactivation of p300 and cellular vulnerability to HDAC inhibition

**DOI:** 10.1038/s41467-023-41245-2

**Published:** 2023-09-02

**Authors:** Longxia Xu, Hongwen Xuan, Wei He, Liang Zhang, Mengying Huang, Kuai Li, Hong Wen, Han Xu, Xiaobing Shi

**Affiliations:** 1 Department of Epigenetics, Van Andel Institute, Grand Rapids, MI USA; 2 Department of Epigenetics and Molecular Carcinogenesis, The University of Texas MD Anderson Cancer Center, Houston, TX USA

**Keywords:** Histone post-translational modifications, Acetylation

## Abstract

The histone acetyltransferase p300/CBP is composed of several conserved domains, among which, the TAZ2 domain is known as a protein-protein interaction domain that binds to E1A and various transcription factors. Here we show that TAZ2 has a HAT autoinhibitory function. Truncating p300/CBP at TAZ2 leads to hyperactive HAT and elevated histone H3K27 and H3K18 acetylation in cells. Mechanistically, TAZ2 cooperates with other HAT neighboring domains to maintain the HAT active site in a ‘closed’ state. Truncating TAZ2 or binding of transcription factors to TAZ2 induces a conformational change that ‘opens’ the active site for substrate acetylation. Importantly, genetic mutations that lead to p300/CBP TAZ2 truncations are found in human cancers, and cells with TAZ2 truncations are vulnerable to histone deacetylase inhibitors. Our study reveals a function of the TAZ2 domain in HAT autoinhibitory regulation and provides a potential therapeutic strategy for the treatment of cancers harboring p300/CBP TAZ2 truncations.

## Abstract (web summary)

The E1A binding protein p300 and its close paralogue CREB-binding protein are transcriptional coactivators with intrinsic histone acetyltransferase activity. Here, the authors show that the TAZ2 domain of p300 has a HAT autoinhibitory function that is relieved upon binding of transcription factors.

## Introduction

**Fig. 1:**
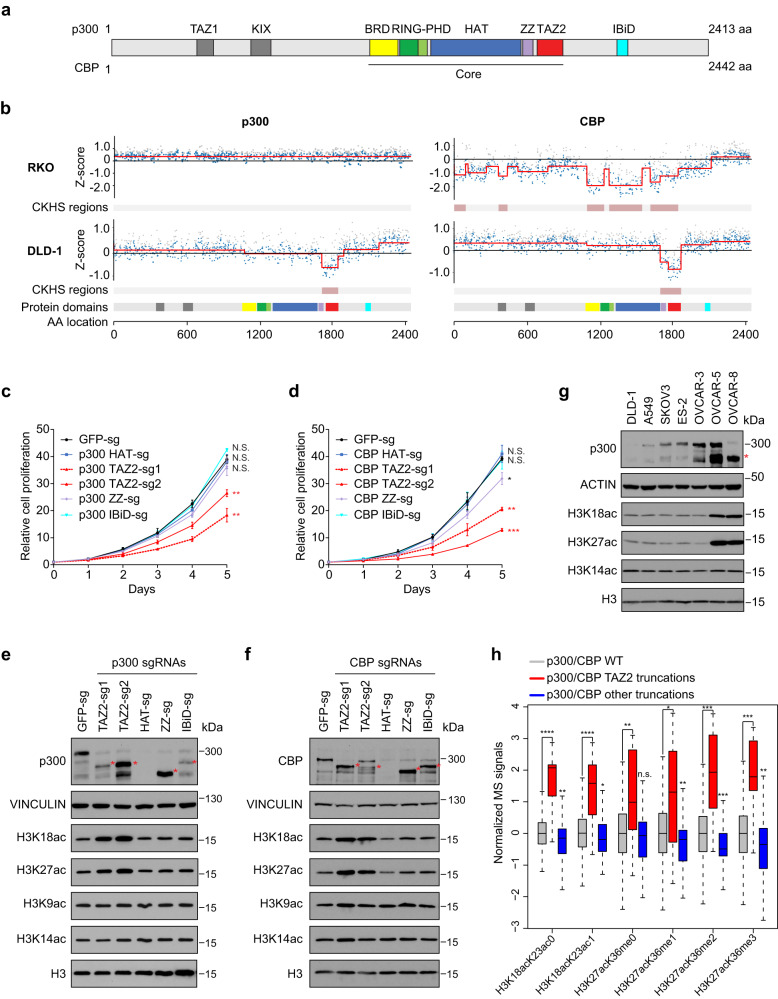
P300/CBP TAZ2 domain truncations inhibit cell growth and increase histone H3K18 and H3K27 acetylation. **a** Schematic of the p300/CBP protein structure. Amino acid numbers, domains, and the catalytic core are labeled. TAZ transcriptional adapter zinc-binding domain, KIX kinase-inducible domain interacting domain, BRD bromodomain, RING Really Interesting New Gene domain, PHD plant homeodomain, HAT histone acetyltransferase domain, ZZ ZZ-type zinc finger, IBiD IRF-3 binding domain. **b** CRISPR-knockout hyper-sensitivity (CKHS) profiles of p300/CBP tiling sgRNA screens in RKO (p300 null, CBP WT) and DLD-1 (p300 and CBP WT) cell lines. X-axis is amino acid location of p300/CBP with domains color-coded as in **a**. Y-axis is Z-score of sgRNA count with negative values indicating dropout effect. Each dot represents an sgRNA mapped to the amino acid location in X-axis. Gray and blue dots represent filtered and remaining sgRNAs, respectively. Red lines show the segmented protein regions and sgRNAs dropout signal levels. **c**, **d** Growth curves of DLD-1 cells expressing sgRNAs targeting the HAT, TAZ2, ZZ, and IBiD domains of p300 **c** and CBP **d**. Error bars represent the standard error of the mean (s.e.m.) of 3 biological replicates. *, **, *** indicate *p*-values < 0.05, *<*0.01, and *<*0.001, respectively. N.S.: not significant (Two-way ANOVA). All p-values are calculated with the GFP-sg as control and are 0.5986, 0.0015, 0.0091, 0.1951 and 0.6126 for p300 HAT-sg, TAZ2-sg1, TAZ2-sg2, ZZ-sg and IBiD-sg, respectively, and 0.9902, 0.0058, 0.0003, 0.036 and 0.9747 for CBP HAT-sg, TAZ2-sg1, TAZ2-sg2, ZZ-sg and IBiD-sg, respectively. **e**, **f** Western blots of p300, CBP, and H3 acetylation in cells as in **c**, **d**. Red asterisks indicate the truncated proteins. Bands below the full-length and truncated p300/CBP proteins are degraded proteins that occurred during sample preparation. **g** Western blots of p300 and histone acetylation in the cell lines containing p300 truncations (OVCAR-5 and OVCAR-8) and p300 WT cell lines (others). Red asterisk indicates truncated p300. In **e**–**g**, blots are representative data from three biological experiments. **h** Boxplot of normalized mass spectrometry signals of H3K18ac and H3K27ac containing peptides from CCLE cell lines with the indicated p300/CBP status. The boxplots are generated by R and show 0, 0.25,0.5, 0.75, and 1 quantile. p300/CBP WT *n* = 1571 cell lines, p300/CBP TAZ2 truncations *n* = 15 cell lines, p300/CBP other truncations *n* = 100 cell lines. *, **, ***, and **** indicate p-values < 0.05, *<*0.01, *<*0.001, and <0.0001, respectively. N.S.: not significant (FDR adjusted Wilcoxon rank sum test). *p*-values from left to right are: 9.76E-5, 1.36E-3, 9.76E-5, 2.25E-2, 7.15E-3, 2.41E-1,1.30E-2, 4.27E-3, 3.20E-4, 1.05E-4, 3.28E-4, 3.99E-3. Source data are provided as a Source Data file.

The E1A binding protein p300 (also known as EP300, lysine acetyltransferase 3B or KAT3B) and its close paralogue CREB-binding protein (CBP, aka CREBBP and KAT3A) are transcriptional coactivators with intrinsic histone acetyltransferase (HAT) activity^1–3^. *P300*/*CBP* are frequently mutated in human cancers, with mutations occurring throughout their coding sequences^4–6^. P300 and CBP are large proteins composed of several conserved domains. The catalytic ‘core’ of p300/CBP consists of HAT and its neighboring domains including the bromodomain (BRD), combined Really Interesting New Gene (RING) domain and plant homeodomain (PHD), ZZ-type zinc finger (ZZ), and the transcriptional adapter zinc-binding domain 2 (TAZ2) (Fig. 1a). Except for TAZ2, all the other domains are known to regulate the HAT activity through multiple intra- and intermolecular interactions^7–12^. For instance, BRD binds to acetylated histones, and this binding is important for substrate histone acetylation by HAT^12,13^. The adjacent RING-PHD module integrates with the HAT substrate-binding pocket and negatively impacts the HAT domain autoacetylation and substrate p53 acetylation^11,14^. Within the HAT domain, there is an unstructured autoregulatory loop (AL, aka autoinhibitory loop or AIL) that serves as an intramolecular ‘pseudo-substrate’ that excludes histones from the active site^15^. Upon HAT activation, AL is subjected to autoacetylation or transacetylation by p300 dimer and expelled from the active site, thus vacating the active site for substrates^15,16^. Acetylated AL is also thought to bind its own BRD, negatively impacting histone acetylation by promoting dissociation of CBP from chromatin^12^. Recently, we identified the ZZ domain of p300 as a reader of the histone H3 tail and we found that H3 binding by ZZ selectively promotes acetylation of histone H3K27 and H3K18^17,18^. The TAZ2 domain was originally discovered as an E1A-binding domain, and it was also shown to bind to the unstructured transactivation domains (TADs) of several transcription factors (TFs) such as p53^19–22^. Thus, TAZ2 is thought to be a protein-protein interaction domain important for TFs to recruit p300/CBP to specific genomic loci. Here we show that the TAZ2 domain has an unexpected function in autoinhibitory regulation of the p300/CBP HAT activity. We propose a model in which TAZ2 cooperates with other HAT neighboring domains to maintain a ‘closed’ state of the HAT active site, an autoinhibition that can be relieved upon TAZ2 binding to the transcription factors.

## Results and discussion

### Truncating p300/CBP at TAZ2 increases histone acetylation

CRISPR tiling screen has emerged as a powerful tool for evaluation of protein domain function in situ^23^. Single guide RNAs (sgRNAs) targeting essential domains generate the strongest lethality phenotypes, thus providing a strategy to identify the protein domains essential for cell survival. To streamline the analysis of CRISPR tiling screens, we developed ProTiler, a computational method for fine-mapping of protein regions that are associated with CRISPR-knockout hyper-sensitivity (CKHS)^24^. To systematically evaluate the functions of the p300/CBP protein domains, we used ProTiler and analyzed p300/CBP CRIPSR tiling screens in RKO and DLD1 cells^25^. RKO is a p300 null colon carcinoma cell line, and DLD1 is a colorectal adenocarcinoma cell line that is wildtype (WT) for p300 and CBP. Because p300 and CBP are functional paralogues, cells are still viable when either p300 or CBP is depleted, but the simultaneous loss of both is lethal^26^. We found that in the p300 null RKO cells, drop-out sgRNAs were enriched in known essential domains of CBP such as HAT and BRD, suggesting that the acetyltransferase activity of CBP is important for RKO cell survival (Fig. 1b). In contrast, in DLD-1 cells that contain WT p300 and CBP, only those sgRNAs targeting the TAZ2 domains were specifically dropped out in the screen, suggesting that the TAZ2 domains are important for DLD-1 cell survival (Fig. 1b). Next, we designed domain-specific sgRNAs to validate the CRISPR tiling screen results. Indeed, expression of either p300 or CBP TAZ2 sgRNAs in DLD-1 cells resulted in strong inhibition of cell proliferation, whereas sgRNAs targeting HAT, ZZ, or the C-terminal IRF-3 binding domain (IBiD) had no or minimal effects (Fig. 1c, d). Importantly, we obtained similar results in three other p300 and CBP WT cancer cell lines, including lung cancer cell lines H1299 and A549, and an ovarian cancer cell line OVCAR-3, in cell proliferation (Supplementary Fig. 1a–f) and competitive cell growth assays (Supplementary Fig. 1g), suggesting a common role of the p300/CBP TAZ2 domains. The CBP ZZ sgRNA showed weak growth inhibition in some cell lines, likely due to the production of a truncated protein with an inert HAT activity^18^ (Fig. 1f and Supplementary Fig. 1k, m), which may act as a dominant negative mutant that also impedes the functions of the full-length p300.

The HAT domain is a critical component of the p300/CBP protein. Targeting the HAT domain by sgRNA-targeted CRISPR-Cas9 editing often leads to destabilization of the entire protein^23^. However, in the current study, sgRNAs targeting the HAT domain did not affect cell survival in the four p300/CBP wild-type (WT) cell lines tested. In contrast, sgRNAs targeting the TAZ2 domain inhibited cell survival. We thus hypothesized that treating cells with sgRNAs targeting TAZ2 results in gain-of-function (GOF) p300/CBP proteins that are harmful to cells. To test this hypothesis, we sequenced the DNA regions near the TAZ2 sgRNA-targeted sites and found that the sgRNAs mainly generated indel mutations that resulted in frameshifts and premature terminations of the proteins in the TAZ2 domain (Supplementary Data 1). Western blot analysis confirmed that TAZ2 sgRNAs caused protein truncations of p300/CBP in all the tested cell lines (Fig. 1e, f and Supplementary Fig. 1j–o). Similarly, sgRNAs targeting the ZZ and IBiD domains also caused protein truncations around their targeted regions, while HAT sgRNAs resulted in knockout of the entire p300/CBP proteins.

We next determined how histone acetylation changed in the cells expressing sgRNAs targeting different domains of p300/CBP. Depleting the p300 protein with the HAT sgRNA had no obvious effects on global histone acetylation levels, due to the compensatory effect of CBP. In contrast, truncating p300 at TAZ2 increased histone acetylation particularly on H3K18 and H3K27, two major p300-acetylating sites on histones (Fig. 1e). Histone acetylation at other sites, such as H3K9 or H3K14, or histone H3 methylation was largely unaffected (Fig. 1e and Supplementary Fig. 1h). The increase in histone acetylation was not due to elevated p300 protein levels, as one of the TAZ2 gRNAs (TAZ2-sg1) resulted in truncated protein expressed at a level lower than that of the endogenous p300 protein in the control cells. Importantly, the increase in histone acetylation was specific to TAZ2 truncations, as truncating p300 at the ZZ or IBiD domain did not affect global histone acetylation levels. Similarly, sgRNAs targeting the TAZ2 of CBP, but not the HAT, ZZ, or IBiD domains, increased global H3K18 and H3K27 acetylation levels (Fig. 1f and Supplementary Fig. 1i). Furthermore, we obtained similar results in H1299, A549, and OVCAR-3 cancer cells (Supplementary Fig. 1j–o). Together, these results suggest that truncating p300/CBP in the TAZ2 domains increases histone H3K18 and H3K27 acetylation in cells.

*P300/CBP* genes are frequently mutated in human cancers and the mutations are scattered throughout the coding sequences, including the TAZ2 domain^4–6^. Ovarian cancer cell lines OVCAR-5 and OVCAR-8 harbor nonsense (C1782*) and frameshift (S1730fs) mutations, respectively, in the p300 TAZ2 domain. Similar to the TAZ2 sgRNA truncation experiments, OVCAR-5 and OVCAR-8 showed higher levels of H3K18ac and H3K27ac compared to p300/CBP WT cell lines such as OVCAR-3, DLD-1, A549, and a few others (Fig. 1g). Furthermore, quantitative mass spectrometric profiling of histone marks from 897 CCLE cancer cell lines^27^ revealed that cell lines with p300/CBP TAZ2 truncations had increased levels of H3K18ac- and H3K27ac-containing peptides relative to p300/CBP WT cells or those with other p300/CBP truncations (Fig. 1h). Collectively, these findings suggest that p300/CBP TAZ2 frameshift or nonsense cancer mutations result in increased histone acetylation, and these alterations may represent the first class of cancer-associated p300/CBP GOF mutations.

### Elevated histone acetylation enhances gene expression

**Fig. 2:**
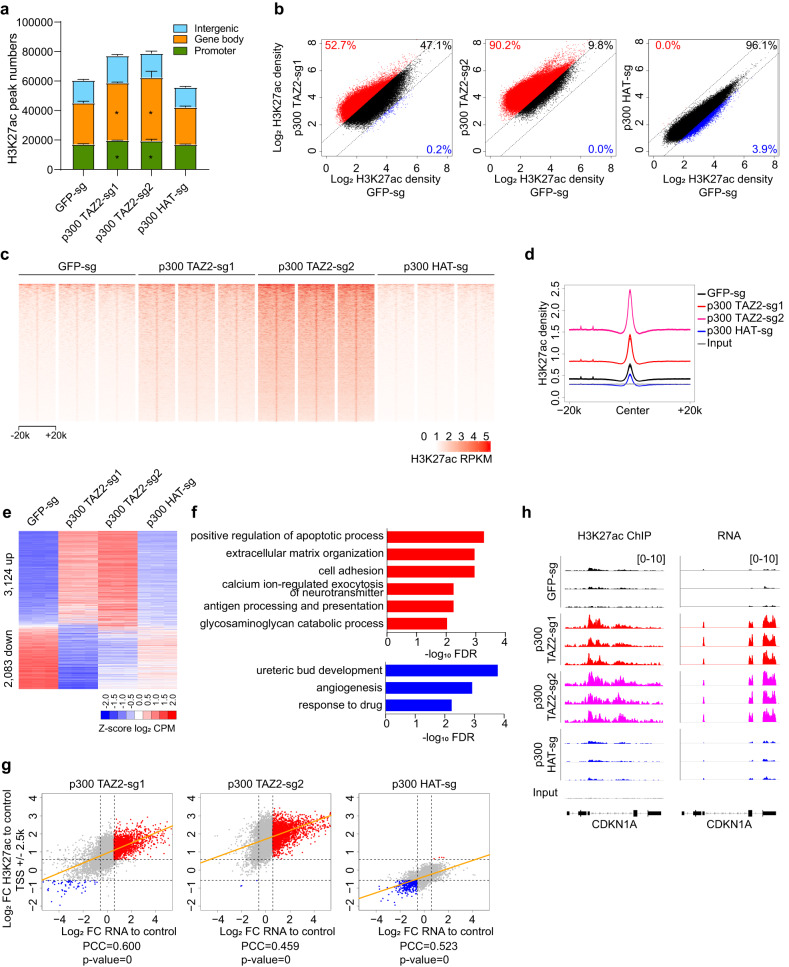
Increased histone acetylation by p300 TAZ2 truncations is associated with enhanced gene expression. **a** Bar plot of H3K27ac ChIP-seq peak numbers in OVCAR-3 cells expressing the indicated p300 sgRNAs and control GFP sgRNA. Error bars represent s.d. of 3 replicates. Asterisks indicate significant increase over peaks in the control cells (Two tail paired t-test, p-value numbers and details in Supplementary Data 2). **b** Dot plots of log_2_ read densities of H3K27ac ChIP-seq peaks from cells expressing p300 TAZ2 (left and middle) and HAT (right) sgRNAs compared to the control cells expressing a GFP sgRNA. Dot values are average of 3 replicates. Red and blue dots indicate relatively high and low H3K27ac signals (FC > 1.5), respectively. **c** Heatmaps of H3K27ac ChIP-seq densities centered on H3K27ac peaks across a ± 20-kb window in cells as in **a**. The color key represents RPKM signal density with darker color representing higher signal. RPKM: Reads Per Kilobase Million. *n* = 3 biological replicates. **d** Average profiles of H3K27ac ChIP-seq densities centered on H3K27ac peaks in cells as in **a**. **e** Heatmap representation of differentially expressed genes in cells as in **a**. Red and blue indicate relatively high and low expression (FC > 1.5 and FDR < 0.05), respectively (details in Supplementary Data 4). CPM: counts per million. *n* = 3 biological replicates. **f** Enriched Gene Ontology (GO) terms (FDR < 0.01) of up and downregulated genes in **e** (details in Supplementary Data 5). **g** Correlation between fold changes of promoter H3K27ac and gene expression. X-axis is log_2_ fold change (FC) of RNA expression and Y-axis is log_2_ fold change of promoter H3K27ac levels in cells expressing p300 TAZ2 (left and middle) and HAT (right) sgRNAs compared to the control cells. Red and blue dots represent genes with increased or decreased H3K27ac and expression (FC > 1.5), respectively. Orange lines indicate fitted linear models. PCC: Pearson correlation coefficient. **h** Integrative Genomics Viewer (IGV) views of H3K27ac ChIP-seq densities and RNA expression of *CDKN1A* in cells as in **a**. Three biological replicates of each cell line are shown. Source data are provided as a Source Data file.

We sought to identify the genomic regions where p300/CBP TAZ2 truncations increased histone H3 acetylation. For this, we performed H3K27ac ChIP-seq experiments in OVCAR-3 and DLD-1 cells expressing p300 TAZ2 sgRNAs, with cells expressing sgRNAs targeting GFP or the p300 HAT domain used as controls. Our analysis identified an average of 60,506 H3K27ac peaks in OVCAR-3 cells expressing the GFP sgRNA, and 55,885 H3K27ac peaks in the HAT sgRNA-expressing cells. In contrast, we identified 77,211 and 78,882 H3K27ac peaks in cells expressing the TAZ2 sgRNAs, which represents an ~30% increase over control cells (Fig. 2a and Supplementary Data 2). Similarly, in DLD-1 cells, we observed an ~45% increase of H3K27ac peak numbers in the p300 TAZ2 truncated cells and an ~25% increase in the CBP TAZ2 truncated cells over the control cells, with strong overlaps between the increased peaks (Supplementary Fig. 2a, e and Supplementary Data 2). The increased H3K27ac marks were broadly distributed across the genome with more increase observed in gene bodies and promoters.

Next, we compared peak density of all the H3K27ac ChIP-seq peaks in control and TAZ2 truncated cells. Our analysis showed that the majority of the H3K27ac peaks (53–90% in OVCAR-3 cells and 46–80% in DLD-1 cells) had higher density in p300 TAZ2 truncated cells, whereas the HAT-sgRNA expressing cells had little difference from the control cells (Fig. 2b and Supplementary Fig. 2b). Heatmaps and average profiles of H3K27ac ChIP-seq densities showed that the increase of acetylation was not only on the H3K27ac peaks, but also in peak flanking regions (Fig. 2c, d and Supplementary Fig. 2c, d). Similar acetylation increases and distribution patterns were also observed when CBP was truncated at the TAZ2 domain in DLD1-cells (Supplementary Fig. 2f–h).

Histone acetylation is normally associated with open chromatin prone to gene activation. We performed ATAC-seq in OVCAR-3 cells and found that almost all the H3K27ac peaks were located within the accessible chromatin regions captured by ATAC-seq (Supplementary Fig. 3a and Supplementary Data 2 and 3). Importantly, the gained H3K27ac peaks in the p300 TAZ2 truncated cells were associated with higher chromatin accessibility compared to the control cells (Supplementary Fig. 3b, c). These results suggest that elevated histone acetylation caused by p300 TAZ2 truncations increases chromatin accessibility, which will likely promote gene expression.

To determine how the global increase in H3K27ac influences gene expression, we performed RNA-seq analysis using the same set of cells used for ChIP-seq and ATAC-seq experiments. We observed that p300 TAZ2 truncations led to dysregulation of ~5,200 genes in OVCAR-3 cells and ~ 2,500 genes in DLD-1 cells, and CBP TAZ2 truncations resulted in dysregulation of ~3,750 genes in DLD-1 cells (Fig. 2e, Supplementary Fig. 4c, f, and Supplementary Data 4). Gene Ontology analysis revealed that the upregulated genes upon TAZ2 truncations were enriched for apoptosis, cell adhesion, and extracellular matrix organization in OVCAR-3 and DLD-1 cell lines, whereas the downregulated genes did not share common terms in these two cell lines (Fig. 2f and Supplementary Data 5). Importantly, the elevated histone acetylation at both promoters and gene bodies in p300 TAZ2 truncated cells showed a strong positive correlation with increased gene expression in both OVCAR-3 and DLD-1 cell lines (Fig. 2g, h and Supplementary Fig. 4a, b, d, e). A similar correlation was also observed when CBP was truncated at the TAZ2 domain in DLD-1 cells (Supplementary Fig. 4g, h). Collectively, these results demonstrate that elevated histone acetylation caused by p300/CBP TAZ2 truncations enhances gene expression.

### TAZ2 inhibits p300 HAT activity

**Fig. 3:**
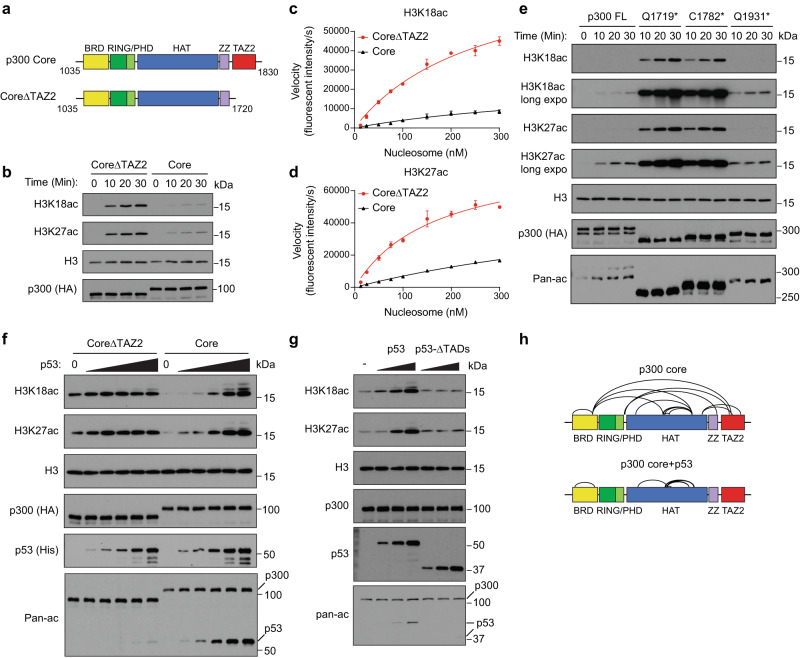
The TAZ2 domain negatively regulates p300 HAT activity. **a** Schematic of p300 Core and CoreΔTAZ2. Domains are color coded as in Fig. 1a. **b** Western blots of in vitro HAT assays using p300 Core and CoreΔTAZ2 proteins purified from 293 T cells. **c**, **d** Kinetic measurement of the catalytic activity of p300 Core and CoreΔTAZ2 on nucleosome. The steady-state velocity was determined based on fluorescent intensity of H3K18ac **c** and H3K27ac **d** per second. The K_m_ of nucleosome substrate (derived from non-linear Michaelis-Menten regression) by Core and CoreΔTAZ2 are 0.82 ± 0.23 μM and 0.17 ± 0.03 μM for H3K18ac, and 0.50 ± 0.14 μM and 0.27 ± 0.04 μM for H3K27ac, respectively. Error bars represent standard error of the mean (s.e.m.) of 3 biological replicates. Representative quantitative Western blots are shown in Supplementary Fig. 5e. **e** Western blots of in vitro HAT assays using the purified full-length p300 protein and truncation mutants identified in human cancers. **f** Western blots of in vitro HAT assays of p300 Core and CoreΔTAZ2 proteins in the presence of increasing amounts of p53 protein. **g** Western blots of in vitro HAT assays of p300 Core in the presence of full-length p53 or p53ΔTADs. Western blots are representative data of 3 **b**, **e**, **g** or 2 **f** biological experiments. **h** Crosslinks (black arcs) identified by CLMS of p300 Core with and without p53 incubation (details in Supplementary Data 6). Source data are provided as a Source Data file.

Because TAZ2 truncation leads to global increase in histone acetylation across several cell types, we suspected that the TAZ2 domain directly regulates p300/CBP HAT catalytic activity. To test this hypothesis, we cloned the p300 catalytic core (referred to as Core, aa 1035–1830) and the truncated Core without TAZ2 (CoreΔTAZ2, aa 1035-1720) and compared their HAT activity (Fig. 3a). We purified the Flag-HA-tagged p300 proteins expressed in 293 T cells (Supplementary Fig. 5a) and performed in vitro HAT assays using unmodified nucleosome as a substrate. P300 Core weakly acetylated histone on H3K18 and H3K27, while CoreΔTAZ2 showed a significantly higher activity acetylating histone (Fig. 3b). Similar results were observed with the CBP Core and CoreΔTAZ2 proteins purified from cells (Supplementary Fig. 5b). Furthermore, domain swapping experiments showed that the CBP TAZ2 domain can effectively inhibit the catalytic activity of the p300 HAT (Supplementary Fig. 5c). To exclude the possibility of contamination from co-purified endogenous HAT enzymes, we expressed the p300 fragments in *E.coli*, and repeated the HAT assays using the purified recombinant proteins (Supplementary Fig. 5d). These data confirmed that CoreΔTAZ2 had higher HAT activity on histones than the Core. A kinetic analysis using quantitative fluorescent Western blot analysis showed that p300 CoreΔTAZ2 acetylated nucleosomes more rapidly and efficiently than the Core on both H3K18 and H3K27 sites (Fig. 3c, d and Supplementary Fig. 5e). These results suggest that the TAZ2 domain has an autoinhibitory role in regulating the HAT activity of p300/CBP.

The TAZ2 domain is composed of four amphipathic helices linked by three zinc-binding loops^20–22,28^ (Supplementary Fig. 5f). To identify the minimal region of TAZ2 required for HAT inhibition, we generated three TAZ2 truncation mutants by deleting α2 through α4 of TAZ2 from the p300 Core, and then compared their HAT activity to that of the Core and CoreΔTAZ2. Deleting the last alpha helix (α4) had little effect on HAT activity, whereas further deleting α3 and α2 gradually increased substrate histone acetylation levels in the HAT assays (Supplementary Fig. 5g). These data suggest that the first three alpha helices are all required for TAZ2-mediated HAT inhibition. Notably, TAZ2 genetic mutations identified in human cancer patients cause various premature terminations of p300/CBP in the TAZ2 domains^4–6^ (Supplementary Fig. 6a). For example, the C1782* found in OVCAR-5 cells terminates p300 in the α3 helix of TAZ2 (Supplementary Fig. 5f). We found that similar to the Q1719* cancer mutant (deprived of the entire TAZ2 domain), the C1782* protein showed a massive increase in autoacetylation and substrate histone acetylation relative to the full-length p300 protein (Fig. 3e and Supplementary Fig. 6b). In contrast, the IBiD truncated cancer mutant Q1931* only showed a weak activity comparable to the full-length p300. In addition to the nonsense mutations, we also tested several missense mutations of TAZ2 found in human cancers. In vitro HAT assays showed that some mutations, such as R1763W, H1767Y, and D1729N, could activate p300 HAT activity, although not as strongly as deleting the entire TAZ2 domain (Supplementary Fig. 6c). On the other hand, mutations that impair TAZ2 DNA binding activity^29^ did not affect TAZ2-medaited HAT inhibition (Supplementary Fig. 6d). Collectively, these data suggest that the first three alpha helices are crucial for TAZ2-mediated HAT-inhibition, and cancer-related mutations in these regions lead to HAT overactivation.

Previous studies have suggested that the α1-α3 helices of TAZ2 interact with the transactivation domains (TADs) of several TFs^15–17^, indicating a potential role of TF binding in modulating TAZ2-mediated HAT inhibition. To investigate this possibility, we performed HAT assays with the p300 Core preincubated with several TAZ2-binding TFs, including p53, p63, FOXM1, C/EBPβ, B-MYB, STAT1, and E2A. Our results showed that, except for E2A, increasing amounts of TFs led to dose-dependent increases in substate histone acetylation by the p300 Core (Fig. 3f and Supplementary Fig. 7a–f), indicating that these TAZ2-binding TFs can active p300. Recombinant E2A protein failed to enhance p300 HAT activity in our assay, possibly due to the lack of the KIX domain in the p300 Core fragment for E2A interaction or proper acetylation of E2A required for its transactivation activity^30,31^. Importantly, TF-mediated p300 HAT activation was specific to the Core that contains the TAZ2 domain, as these TFs did not activate the CoreΔTAZ2 protein. Furthermore, the TADs were required for HAT activation, as deletion of the TADs abrogated p53-mediated HAT activation (Fig. 3g). Together, these findings suggest that relief of TAZ2-mediated HAT inhibition likely represents a new paradigm for transcriptional activation through p300/CBP.

To determine how TF binding activates p300 HAT activity, we performed crosslinking mass spectrometry (CLMS) analysis of the p300 Core with and without p53 incubation. Without p53, multiple crosslinked peptides between TAZ2 and the HAT, PHD, and BRD domains were detected by mass spectrometry (Fig. 3h and Supplementary Data 6), indicating that TAZ2 likely cooperates with these domains to maintain a closed, inactive state of the HAT module. Indeed, superimposition of the AlphaFold predicted p300 structure and a recent cryo-EM structure of p300 CoreΔTAZ2 in complex with nucleosome^32^ shows that TAZ2 is in proximity to HAT and neighboring domains, preventing nucleosome on the opposite side of HAT from getting access to the HAT substrate binding grove (Supplementary Fig. 7g). Importantly, adding p53 diminished the inter-domain crosslinked peptides (Fig. 3h), suggesting that p53 binding dissociates TAZ2 from HAT, promoting the HAT module to switch from a closed state to an open state ready for substrate acetylation (Supplementary Fig. 7h).

### Cells with TAZ2 truncations are vulnerable to HDAC inhibitors

**Fig. 4:**
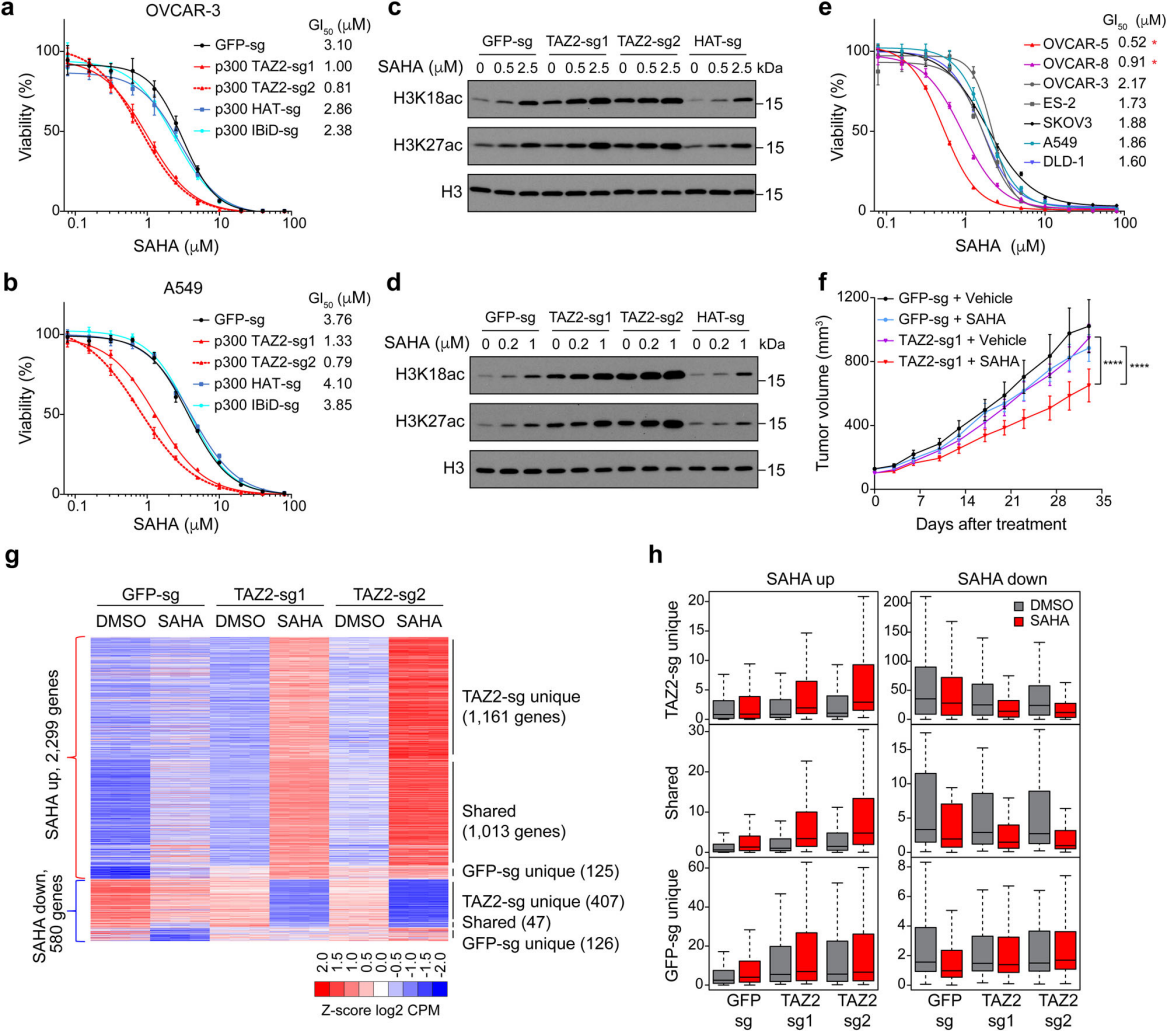
Cells containing p300/CBP TAZ2 truncations are vulnerable to HDAC inhibitors. **a**, **b** Cells with p300 TAZ2 truncation are sensitive to SAHA. Cell viability was measured in OVCAR-3 **a** and A549 **b** cells expressing p300 TAZ2 and HAT sgRNAs or GFP sgRNA and treated with different doses of SAHA for 3 days. Data are representative of 3 biological experiments and presented as mean ± s.d. GI_50_, the drug concentration for 50% of maximal inhibition of cell proliferation. **c**, **d** Western blots showing SAHA-induced histone acetylation changes in cells as in **a**, **b**. Blots are representative of 2 biological experiments. **e** Cancer cell lines containing p300 TAZ2 truncations are more sensitive to SAHA. Cell viability measurement for p300/CBP WT cell lines and the two cell lines (OVCAR-5 and OVCAR-8, indicated by asterisks) containing p300 TAZ2 truncation mutations. Data are representative of 3 biological experiments and presented as mean ± s.d. **f** Tumor growth in xenografted A549 cells expressing p300 TAZ2 sgRNA1 or GFP sgRNA under SAHA treatment (25 mg/kg/day, daily IP). Data presented are mean ± s.e.m. of *n* = 10, 10, 9, 10 mice (from top to bottom). ****P < 0.0001 (Two-way ANOVA). The p-value of GFP-sg SAHA treatment compared to Vehicle is 0.0755, the p-value of TAZ2-sg1 SAHA to Vehicle *<*0.0001, the p-value of TAZ2-sg1 SAHA to GFP-sg SAHA < 0.0001 (Two-way ANOVA). **g** Heatmap of SAHA-induced up and downregulated genes in A549 cells expressing p300 TAZ2 and GFP sgRNAs and treated with DMSO and SAHA (0.5 µM) for 24 h. Red and blue indicate relatively high and low expression (FC > 1.5 and FDR < 0.05), respectively (details in Supplementary Data 7). CPM: counts per million. *n* = 3 biological replicates each group. **h** Box plots of averaged CPM values of SAHA-induced expression of gene groups as in **g**. DMSO treated samples are in gray and SAHA treated samples in red. Boxplots are generated by R and show 0, 0.25,0.5, 0.75, and 1 quantile. Gene numbers in each group are the same as presented in **g**. DMSO treated samples are in gray and SAHA treated samples in red. Source data are provided as a Source Data file.

The global increase in histone acetylation caused by p300 TAZ2 truncations is reminiscent of the effect of histone deacetylase (HDAC) inhibition by specific or pan-HDAC inhibitors. HDAC inhibitors are known to induce histone and protein hyperacetylation, resulting in gene expression changes that lead to various antitumor responses, including differentiation and cell death^33^. As excessive acetylation can be toxic to cells, we reasoned that due to the higher basal levels of histone acetylation, cells harboring p300/CBP TAZ2 truncations might be more sensitive to HDAC inhibitors. To test this, we truncated the p300 proteins in OVCAR-3 and A549 cells with TAZ2 sgRNAs, treated the cells with the FDA-approved HDAC inhibitor Vorinostat (aka Suberoylanilide hydroxamic acid, or SAHA), and measured cell survival. We found that cells expressing the p300 TAZ2 sgRNAs were 3-4 times more sensitive to SAHA than control cells (Fig. 4a, b). In contrast, depleting p300 protein with the HAT or IBiD sgRNAs did not affect the SAHA GI_50_. Western blot analysis showed that SAHA induced higher levels of histone hyperacetylation in cells expressing p300 TAZ2 sgRNAs (Fig. 4c, d). Similar results were obtained with another FDA-approved HDAC inhibitor, Panobinostat (Supplementary Fig. 8a–d). Truncating CBP TAZ2 in OVCAR-3 and A549 cells also resulted in increased cell death upon SAHA and Panobinostat treatment (Supplementary Fig. 8e–h). Furthermore, compared to the p300/CBP WT cell lines, cancer cell lines with intrinsic p300 TAZ2 truncations (OVCAR-5 and OVCAR-8) showed higher sensitivities to HDAC inhibitors, but not p300/CBP inhibitors A-485 or CBP-30 (Fig. 4e and Supplementary Fig. 8i–k). Together, these results demonstrate that cells with p300/CBP TAZ2 truncations are vulnerable to HDAC inhibitors.

Next, we aimed to evaluate the efficacy of HDAC inhibitors in killing tumors with p300/CBP TAZ2 truncations in vivo. For this xenograft experiment, we used A549 cells as they were found to graft better in mice than OVCAR-3 cells. We transplanted A549 cells expressing a p300 TAZ2 sgRNA and a control GFP sgRNA into athymic nude mice and allowed the xenografted tumors to reach the volume of ~150 mm^3^ before drug treatment. Subsequently, we treated the tumors with a low dose of SAHA (25 mg/kg/day) for 5 weeks. SAHA inhibited tumor growth in the xenografted cells containing p300 TAZ2 truncation, whereas it had little effect on tumor growth in the xenografted control cells (Fig. 4f). Importantly, although the TAZ2 sgRNA-expressing cells contained mostly truncated p300 protein at transplantation, after 5 weeks of SAHA treatment, most of the survived cells in the xenografted tumors contained WT p300 (Supplementary Fig. 9a, b), indicating that the cells containing p300 TAZ2 truncations were outcompeted by the residual p300 WT cells in the tumor. We also generated isogenic A549 cells bearing p300 TAZ2 truncation by selecting CRISPR-edited single cells and repeated the SAHA treatment experiments using xenografted tumors (Supplementary Fig. 9c–f). The results further corroborated that p300 TAZ2 truncation renders cells vulnerable to HDAC inhibition in vivo.

To determine how SAHA suppressed tumor growth of the cells containing p300 TAZ2 truncation, we carried out RNA-seq analysis to identify global gene expression changes in control and the p300 TAZ2 truncated A549 cells with and without SAHA treatment. Treatment with low dose of SAHA (0.5 μM) induced ~2,600 genes to be up or downregulated in cells expressing the p300 TAZ2 sgRNAs, whereas only about half of the number genes were dysregulated in the control cells (Fig. 4g, Supplementary Fig. 9g and Supplementary Data 7). Notably, SAHA induced much larger changes in gene expression in the p300 TAZ2 truncated cells compared to the control cells (Fig. 4h), suggesting that the higher basal levels of histone acetylation in the p300 TAZ2 truncated cells prime the cells to be more susceptible to HDAC inhibitors. Gene ontology analysis also indicated that SAHA-treatment induced a similar set of genes and pathways as did p300 TAZ2 truncation, and that the combined treatment (p300 TAZ2 truncation + SAHA) showed a strong synergy in gene induction (Supplementary Fig. 9h, i, and Supplementary Data 8). Collectively, these results suggest that HDAC inhibitors reinforce the effects of global histone hyperacetylation caused by p300/CBP TAZ2 truncations, providing a potentially effective treatment option for cancers harboring p300/CBP TAZ2 truncations.

In this study, we have discovered a novel role of the TAZ2 domain of p300/CBP in the negative regulation of the HAT activity. Previous studies have identified two motifs, AL and RING finger, within the p300 core that negatively regulate the HAT activity. AL was thought to act as an autoinhibitory loop that blocks the HAT active site from the histone substrates^11,15,34^, while the RING finger is positioned over the HAT substrate-binding pocket, hindering the access of AL and substrates^11^. However, other studies have suggested that deletion of AL or the RING finger in p300 or CBP impairs substrate histone and p53 acetylation instead^12,14,16^. Therefore, the actual roles of AL and RING in HAT regulation are likely context dependent. On the other hand, our findings reveal that truncations of TAZ2 in full-length p300/CBP and the core fragments greatly enhance the catalytic activity of HAT, both in vitro and in cells, demonstrating that TAZ2 is a bona fide negative regulator of HAT.

During the course of this study, two studies reported that the BRD4-NUT fusion protein contains two TADs in NUT that bind to TAZ2, and the TAD-TAZ interaction triggers allosteric activation of p300 and acetylation-driven liquid-like condensation on chromatin^35,36^. Ibrahim et al. showed that in vitro, the NUT TADs stimulate autoacetylation of the p300s fragment (aa324-2094), but not a shorter p300 fragment (aa1048-1664)^36^. However, as compared with the p300s fragment, the shorter p300 fragment lacks several functional domains including TAZ1, KIX, TAZ2, and IBiD and the intrinsically disordered regions between them, thus it remains unclear whether the other domains beside TAZ2 also contribute to NUT-mediated HAT activation. In the current study, we have directly compared the p300 Core fragments with and without TAZ2 in vitro and in cells, providing strong and direct evidence demonstrating that the TAZ2 domain has a HAT autoinhibitory function. Moreover, Ibrahim et al. mainly monitored p300 autoacetylation in their study^36^, whereas our study has extended the evaluation of p300 HAT activation by assessing substrate histone acetylation on multiple histone residues primarily acetylated by p300. Additionally, p300 has been reported to bind with numerous TFs via TAZ2-TAD interactions^7,8^. In our study, we tested seven TFs and found that six of them activate p300 in a TAZ2-dependent manner, indicating that relief of TAZ2-mediated HAT inhibition by TFs likely represents a previously unrecognized mechanism for transcriptional activation through p300/CBP. Collectively, these findings suggest that TAZ2 inhibits HAT, at least in part, by modulating p300 autoacetylation and that this autoinhibition is relieved upon TAZ2 binding to transcription factors, leading to an active p300 for substrate (such as histones) acetylation (Supplementary Fig. 7h).

*P300/CBP* are among the most frequently mutated genes in human cancers. Genetic mutations of p300/CBP are scattered across their coding sequences, particularly in the HAT domain. Many of the HAT domain mutations are loss of function (LOF) mutations that impair the catalytic activity, suggesting that p300/CBP function as tumor suppressors^37^. P300/CBP have also been reported to have a pro-oncogenic activity in some cancer types, such as prostate cancer, in part through their transcriptional coactivator function^38,39^. In this study, we find that p300 TAZ2 truncations result in HAT hyperactivation and increased global histone acetylation in cells, suggesting a GOF of the HAT activity. Interestingly, we find that cells containing p300 TAZ2 truncations are vulnerable to HDAC inhibition. Low doses of HDAC inhibitors selectively kill cells containing p300/CBP TAZ2 truncations without affecting the p300/CBP wildtype cells. Given the frequency of p300/CBP mutations in human cancers and the presence of TAZ2 mutations in various cancer types, our findings provide a promising therapeutic strategy for treating cancers with p300/CBP TAZ2 truncations.

## Methods

### Animal work

Research conducted in this paper complies with all relevant ethical regulations at Van Andel Institute. All animal experiments were performed under the protocol approved by the Institutional Animal Care and Use Committee (IACUC) of Van Andel Institute.

### Materials

Human full-length (FL) p300 cDNA was cloned in pENTR3C and subsequently cloned into pCDH-3Flag-HA destination vectors using Gateway techniques (Invitrogen). The cDNA encoding the Core (aa1035-1830) and CoreΔTAZ2 (aa1035-1720) of p300 were cloned into pCDH-3Flag-HA and pGEX-6p-1 (Novagen). P300 truncations and point mutations were introduced to the coding sequences in the context of FL or p300 Core using site-directed mutagenesis kit (Stratagene). Cbp Core (aa1072-1868) and CoreΔTAZ2 (aa1072-1759) were amplified from pcDNA3β-FLAG-CBP-HA plasmid (Addgene) and cloned into pCDH-3Flag-HA. FL p53 (aa1-393) and p53ΔTAD (aa62-393) were cloned into pET21a vector. FL C/EBPβ (aa1-345) and FOXM1 (aa1-762) were cloned into pET19b vector. B-MYB (aa1-700), E2A (aa1-654), STAT1 (aa1-750) and p63α (aa1-641) were cloned into modified pET28a vector with C terminal His-tag. Migr-Notch1-L1601P∆PEST plasmid was gifted from Dr. Adolfo Ferrando, Columbia University Medical Center. Antibodies used in this study are provided in Supplementary Table 1.

SgRNAs used in this study include *GFP* sgRNA: GGGCGAGGAGCTGTTCACCG; non-targeting control sgRNA: GAGGAGCAAGGACTAAAGCC; *p300* TAZ2 sgRNA1: CTTGGCATGGTAGCA-GCAGA; *p300* TAZ2 sgRNA2: AACCCGCTTCATCTTCTGGC; *p300* HAT sgRNA: GAATCACCCTGAGTCAGGAG; *p300* ZZ sgRNA: AGCACCATGTGGAGACACGC; *p300* IBiD sgRNA: AGCGGAGACGCAGCGCCAGA; CBP TAZ2 sgRNA1: GCGGGTGGTGCAGCACAC-CA; *CBP* TAZ2 sgRNA2: CTTGGCGTGGTAGCAGCAGA; *CBP* HAT sgRNA: AGCCAAAACGA-CTGCAGGAG; *CBP* ZZ sgRNA1: CAACGAGTGCAAGCACCACG; *CBP* ZZ sgRNA2: AGCACCACGTGGAGACGCGC; *CBP* IBiD sgRNA: CTGCAGCGGTGGAAGCGGCT. *TP53* sgRNA: CCATTGTTCAATATCGTCCG.

### P300/CBP tiling-sgRNA screen analysis

The tiling-sgRNA screens of p300/CBP in DLD-1 and RKO cells were retrieved from the Munoz dataset^25^ and the protein regions associated with CRISPR-knockout hyper-sensitivity (CKHS) were called using ProTiler^24^. Two major steps were used in CKHS calling: outlier adjustment and signal segmentation. We set two parameters for outlier adjustment as default (f = 5, t1 = 2), where f represents the half size of sliding window and t1 represents the threshold to determine the outliers among the signals within the sliding window. Considering the large size of the proteins, a more relaxed tuning parameter (t2 = 2, default 1.5) was set in the signal segmentation to exclude trivial segments caused by remaining outliers. ProTiler software can be downloaded from https://github.com/MDhewei/protiler.

### Cell culture and virus transduction

DLD-1 and H1299 cell lines were purchased from ATCC and cultured in RPMI1640 (Corning). A549 and HEK293T cell lines were purchased from ATCC and cultured in DMEM (Corning). OVCAR-3 and OVCAR-8 cells were gifted from Dr. Jose Teixeira at Michigan State University and cultured in DMEM/F-12 (Corning). OVCAR-5 was gifted from Dr. Xiongbin Lu at Indiana University School of Medicine and cultured in DMEM (Corning). ES-2 and SKOV3 were gifted from Dr. Hui Shen at Van Andel Institute and cultured in Mccoy’s 5 A (Thermo Fisher Scientific). All the cell lines were originally from commercial suppliers such as ATCC and Sigma. All media were supplemented with 10% fetal bovine serum (Sigma), 1 mM sodium pyruvate (Corning), 1% non-essential amino acids (HyClone), and 1% penicillin–streptomycin (Corning). Immortalized HBEC cells were cultured in Keratinocyte SFM (Thermo Fisher Scientific) supplemented with 50 μg/ml bovine pituitary extract (BPE; Thermo Fisher Scientific) and 5 ng/ml EGF (Thermo Fisher Scientific). All gifted cell lines were authenticated by STR profiling at MSU and MD Anderson Cytogenetics and Cell Authentication Core and tested for mycoplasma contamination.

Individual sgRNAs were cloned into pLentiCRISPR v2 or pLentiCRISPR V2-Blast (Addgene) vectors and transfected into 293 T cells together with psPAX2 and pMD2.G plasmids at a 2:2:1 ratio using the X-TremeGENE HP DNA transfection reagent (Sigma). Virus supernatant was collected and filtered through a 0.45 μm syringe filter 48 h post transfection. For cell transduction, 0.5-1 × 10^6^ cells were seeded in 6-well plate 24 h before transduction. Cells were transduced with 3 ml lentivirus containing 4μg/ml polybrene by spinoculation at 1100 g, RT for 1 h. After 3 h incubation at 37 °C, medium containing virus was removed and replaced with fresh medium. Cells were selected with 2 μg/ml puromycin or 10 μg/ml Blasticidin (Thermo Fisher Scientific) for 4-6 days.

### Cell viability measurement and drug treatment

DLD-1, H1299, and A549 cells (500) or OVCAR-3 cells (1000) were seeded in 96-well plates and cultured for 4-6 days. Cell viability was measured using the CellTiter-Glo luminescent cell viability assay kit (Promega) according to the manufacturer’s instruction. For Vorinostat (SAHA) and Panobinostat (LBH589) treatment, 2000 cells were seeded in 96-well plates in 100ul medium 24 h before treatment. Cells were treated with SAHA, Panobinostat, or 0.1% DMSO for 3 days before cell viability measurement. Cell viability was calculated as % of survived drug-treated cells relative to DMSO treated cells. For gene expression analysis of SAHA treatment, A549 cells were treated with SAHA (0.5 µM) or DMSO (0.1%) for 24 h before RNA was collected for RNA-seq analysis. For competitive proliferation assay, OVCAR-3 cells were transduced with sgRNAs cloned in lentiCRISPR V2-mCherry vector and analyzed for mCherry expression 3 days later. The percentage of sgRNA-expressing cells (mCherry^+^) was measured over time using flow cytometry.

### Western blot analysis

Cells were washed with PBS and lysed with 1X SDS lysis buffer containing 50 mM Tris-HCl pH6.8, 2% SDS, and 6% glycerol. Samples were boiled at 95 °C for 15 min. Protein concentrations were measured using the *DC* protein assay kit (Bio-Rad) and adjusted to 1 mg/ml with SDS lysis buffer containing 1% β-mercaptoethanol and 0.01% bromophenol blue of final concentrations. Samples were boiled for another 5 min before subjected to Western blotting. For proteins extracted from tumors, tumors were cut into small pieces and disrupted by grinding in liquid nitrogen. The grinded powders were dissolved in SDS lysis buffer and sonicated with 10% output, 20ss on, 80ss off for 5 s. Samples were boiled at 95 °C for 10 min and supernatants were collected for Western blot analysis. Information of all antibodies used is provided in Supplementary Table 1.

### Protein expression and purification

p300 Core (aa1035-1830) and p300 CoreΔTAZ2 (aa1035-1720) constructed in the pGEX-6p-1 vector were transformed in Rosetta™2(DE3) pLysS competent cells (Novagen) and the GST-tagged proteins were induced with 0.4 mM IPTG at 16 °C for 20 h in 2YT medium supplemented with 100 mM ZnCl_2_. Cell pellets were resuspended in Buffer 1 (50 mM Tris-HCl pH7.5, 300 mM NaCl, 1.5 mM MgCl_2_, 1 mM EDTA, 1% Triton100, 5 μM ZnCl_2_, 10% glycerol, 1 mM PMSF, and 1x cOmplete™ EDTA-free Protease Inhibitor Cocktail) and lysed using sonication or homogenizer. The lysates were centrifuged at 12,000 g for 30 min, and the supernatants were incubated with Glutathione Sepharose® 4B beads (Sigma) at 4° for 2 h. Beads were then washed with Buffer 2 (50 mM Tris-HCl pH7.5, 300 mM NaCl, 1.5 mM MgCl_2_, 0.2 mM EDTA, 0.1% NP40, 5 μM ZnCl_2_, 10% glycerol, and 1 mM PMSF) for three times and GST-tagged proteins were eluted with elution buffer containing 50 mM Tris-HCl pH7.5, 300 mM NaCl, 10% glycerol, and 15 mg/ml GSH. Full-length p53 and p53ΔTAD (aa62-393) constructed in the pET21a vector were transformed in Rosetta™2(DE3) pLysS competent cells (Novagen) and the His-tagged proteins were purified using Ni-NTA resins following the manufacture’s instruction. The eluted proteins were dialyzed in buffer containing 50 mM Tris-HCl pH7.5, 100 mM NaCl, 1 mM DTT and 10% glycerol to remove imidazole.

For flag-tagged protein purification, p300 full-length (aa1-2414), the p300 Core (aa1035-1830), p300 CoreΔTAZ2 (aa1035-1720), p300 Core with CBP-TAZ2 (p300 aa1035-1724 fused with CBP aa1763-1867), and the p300 TAZ2 truncation mutants in the pCDH-3Flag-HA vector were expressed in 293 T cells for 2 days. Cells were lysed in cell lysis buffer (50 mM Tris-HCl pH7.5, 300 mM NaCl, 1.5 mM MgCl_2_, 1 mM EDTA, 1% Triton100, 10% glycerol, 1 mM PMSF, and protease inhibitors) and sonicated briefly. The cell lysates were incubated with anti-Flag M2 beads (Sigma) at 4° for 4 h. The beads were then washed three times with buffer (50 mM Tris-HCl pH7.5, 500 mM NaCl, 1.5 mM MgCl_2_, 0.2 mM EDTA, 0.1% NP40, 10% glycerol, and 1 mM PMSF) and eluted with 100ul of elution buffer (50 mM Tris-HCl pH7.5, 300 mM NaCl, 1 mM DTT, 10% glycerol, and 0.4 mg/ml Flag peptide) at RT for 30 min.

### In vitro histone acetyltransferase (HAT) assay

Purified p300 and CBP proteins (50 nM) were mixed with recombinant human mono-nucleosomes (100 nM, EpiCypher) in 50 μL of HAT assay buffer containing 50 mM Tris pH8.0, 100 mM NaCl, 1 mM DTT, 10% glycerol and cOmplete™ EDTA-free Protease Inhibitor. The mixtures were preincubated at 37 °C for 5 min before adding 100 mM Acetyl-CoA and incubation for 10-, 20-, or 30-min. Reactions were stopped by adding 2xSDS gel loading buffer and boiling at 95 °C. For p53 competition assays, different amounts of p53 were incubated with p300 at RT for 10 min before adding nucleosomes. Samples were analyzed by SDS-PAGE and Western blot analysis. For fluorescent Western blots, signals were detected by ChemiDoc MP Imaging System and quantified by Image Lab.

### Crosslinking mass spectrometry (CLMS)

The GST-tagged p300 Core protein was purified through affinity purification using the GST HiTrap column (Sigma) followed by ion-exchange on HiTrap SP Sepharose FF column (GE Healthcare). The protein was washed with buffer containing 20 mM HEPES pH 7.0, 50 mM KCl, 0.5 mM TCEP, and 10% glycerol, and eluted with the same buffer containing 300 mM KCl. Fractions of peak center were collected and used for crosslink. His-tagged full-length p53 was purified by affinity purification using Ni-NTA resins followed by gel filtration on HiLoad 16/600 Superdex 200 column (GE Healthcare). The column was equilibrated with 20 mM HEPES, pH8.0, 300 mM KCl, 0.5 mM TCEP and 10% glycerol, and the p53 proteins at peak center were collected. For p53 competition, 115 μg of the p300 Core protein was mixed with 207 μg of p53 (at the molecular ratio of 1:4) and incubated at RT for 10 min before adding 1 mM DSSO for crosslinking. Reactions were quenched by adding 20 mM NH_4_HCO_3_. After reduction by 2 mM DTT and alkylation by 4 mM chloroacetamide, samples were digested with 2% Trypsin (Promega) at 37° for 20 h and quenched by adding 0.1% formic acid. After C18 tips desalting, peptides were analyzed by mass spectrometry at the UCI High-end Mass Spectrometry Facility. Data were processed by ProteoWizard MSConvert (v. 3.0.10738) and Protein Prospector (v.5.19.1).

### ChIP and ChIP-seq analysis

ChIP was performed essentially as described previously^40^. Briefly, cells (5-10 × 10^6^) were crosslinked by 1% formaldehyde in PBS for 10 min and stopped by adding 125 mM glycine for 5 min at room temperature. Nuclei were isolated using cell lysis buffer (5 mM PIPES pH 8.0, 85 mM KCl, 1% NP-40, and protease inhibitors) for 20 min at 4 °C. The isolated nuclei were resuspended in nuclei lysis buffer (50 mM Tris, pH 8.0, 10 mM EDTA, and 1% SDS) and sonicated for 800 s using Covaris E220 Evo. For H3K27ac ChIP, *Drosophila* chromatin spike-in were added to 1% of total chromatin. The sonicated chromatin samples were incubated with corresponding antibodies at 4 °C overnight. Dynabeads™ Protein G (Thermo Fisher Scientific) were added and incubated for 1 h, and washed twice with low salt wash buffer (20 mM Tris pH 8.0, 150 mM NaCl, 2 mM EDTA, 1% Triton X-100, and 0.1% SDS), twice with high salt wash buffer (20 mM Tris pH 8.0, 500 mM NaCl, 2 mM EDTA, 1% Triton X-100, and 0.1% SDS), once with LiCl wash buffer (20 mM Tris pH 8.0, 250 mM LiCl, 1 mM EDTA, 1% NP-40, and 1% Na-Deoxycholate), and once with TE buffer (10 mM Tris, pH 8.0, 1 mM EDTA). Bound DNA was eluted using fresh 50 mM NaHCO3 and 1% SDS, reverse crosslinked and purified using PCR purification kit (Qiagen).

ChIP-seq libraries were constructed using the KAPA Hyper Prep Kit (Roche) and sequenced by Illumina NovaSeq 6000 at the Van Andel Institute Genomics Core. Fastq reads were mapped to hg38 human genome and dmel_r6.24 drosophila genome (only for spike-in) by HISAT2 (v2.1.0)^41^ with –no-spliced-alignment -k 1 -X 1000. Spike-in adjust ratios were calculated as (mapping ratio_GFP sg_)/(mapping ratio_Sample_). Peaks were called by MACS2 (v2.1.2)^42^ with –broad against input. H3K27ac peaks were separated into promoter (±2.5 kb around TSS), gene body (2.5 kb downstream of TSS to TTS), and intergenic regions (peaks not on promoter or gene body), and paired t-tests were performed cross each biological replicate. Intersections between peaks were generated by bedtools intersect (v2.27.1). To get unions between peaks, bed files were process through the pipeline cat | bedtools sort | bedtools merge. Wig files were generated by Danpos2 (v2.2.2)^43^. BigWig files were transformed from wig files with wigToBigWig (v4). Heatmaps and profiles of merged H3K27ac peaks were generated by Danpos2. Heatmaps were visualized by ggplot2 and reshape2 libraries in R (v3.5.1). Tracks were visualized by IGV (v2.7.2)^44^. Pearson correlation coefficient (PCC) and p-values between fold change of promoter or enhancer H3K27ac and gene expression, and fitted linear models were calculated by cor.test function and lm function of R (v3.5.1), respectively.

### ATAC-seq analysis

OVCAR3 cells (1 × 10^5^) were collected and processed using the ATAC-Seq Kit (Active Motif) following the manufacturer’s instructions. Libraries were sequenced by Illumina NovaSeq 6000 for 50 bp pair-end reads. Fastq reads were mapped to hg38 human genome by HISAT2 (v2.1.0)^41^. Samtools (v1.9) were used to remove mitochondrial DNA and duplicates reads (samtools markdup -r). ATAC-seq peaks were called by MACS2 (v2.1.2)^42^ with -f BAMPE -q 0.01. Intersections between peaks were generated by bedtools intersect (v2.27.1). Venn diagrams of OVCAR-3 H3K27ac peaks and ATAC-seq peaks were generated by Venn Diagram Maker Online (https://www.meta-chart.com/venn). Significances of overlapping were calculated by overlapPermTest function in regioneR library^45^ with ntimes = 5000. Profiles of both H3K27ac ChIP-seq and ATAC-seq densities on gain or loss H3K27ac peaks were generated by Danpos2.

### RNA isolation and RNA-seq analysis

Total RNA was extracted from OVCAR-3, DLD-1, and A549 cells using RNeasy Plus Mini Kit (Qiagen). RNA-seq libraries were prepared using KAPA RNA HyperPrep Kit with RiboErase (HMR) (Roche) or KAPA Stranded mRNA-Seq Kit (Roche) following the manufacturer’s instructions and sequenced by Illumina NovaSeq 6000. Fastq reads were mapped to hg38 human genome by HISAT2 (v2.1.0)^41^ with -k 1. CPM (counts per million) and fold change values were calculated by HTSeq (v0.11.3)^46^ with –stranded=no -a 0 and edgeR (v3.16.5)^47^ with Trimmed Mean of M-values (TMM) and Exact test model. differentially expressed genes (DEGs) were filtered by FDR < 0.05 and FC > 1.5. Heatmaps of OVCAR-3 and DLD-1 RNA-seq data presents union of both up and down regulated DEGs from 2 TAZ2 sgRNAs, genes were ranked by its fold change of average TAZ2 sgs vs. GFP sg from high to low, z-score normalization was performed on each row. Gene Ontology biological processes term enrichment was done by DAVID 6.8^48,49^. Wig files were generated by Danpos2 (v2.2.2)^43^. BigWig files were transformed from wig files with wigToBigWig (v4). Tracks were visualized by IGV (v2.7.2)^44^. Venn diagrams of A549 DEGs were generated by Venn Diagram Maker Online (https://www.meta-chart.com/venn). Fisher’s exact test between DEGs in Venn diagrams are calculated by fisher.test function in R (v3.5.1). Heatmap of A549 RNA-seq data presents six classes of genes highlighted in Venn diagrams, z-score normalization was performed on each row. The corresponding box plot was generated from raw CPM values in each class of genes by boxplot library in R (v3.5.1). The bubble plots of GO enrichment were generated by Microsoft Excel.

### Mouse xenograft and drug treatment

All animal experiments were performed under the protocol approved by the Institutional Animal Care and Use Committee (IACUC) of Van Andel Institute. Mice were housed in 12 h light cycle (7am–7pm) with 72F0 and 50% humidity. A549 cells (4 × 10^6^) stably expressing p300 TAZ2 sgRNA1 or GFP sgRNA, or TAZ2 truncated isogenic A549 cells selected from single clone, were resuspended in PBS, mixed with 50% growth factor reduced Matrigel (Corning), and injected subcutaneously on the right flank of 6-8 weeks old female athymic nude mice (Jackson laboratory, strain #007850). When tumors reached 100–150 mm^3^, mice were randomly assigned to vehicle (DMSO) or SAHA (Vorinostat, Selleckchem) treatment group. SAHA was dissolved in DMSO, diluted into 10% (2-hydroxypropyl)-β-cyclodextrin (Sigma), and administered through intraperitoneal (i.p.) injection at a concentration of 25 mg/kg body weight. Mice were subjected to once daily treatment for up to 35 days. Tumors were measured by calipers twice a week. Animals were euthanized when tumors reached 15 mm in diameter or if animals became moribund, as per institutional IACUC guidelines. Tumor volume was calculated using (Length × Width^2^)/2 and plotted as mean (volume in mm^3^) ± s.e.m. with statistics by two-sided Student’s t-test at each timepoint. Tumor growth curve kinetics were analyzed by two-way ANOVA.

### Statistical analysis

Experimental data were analyzed using Prism 9 software (GraphPad) by two-way ANOVA or two-sided Student’s t-test. *P-*values  <  0.05 are considered statistically significant unless stated otherwise. Statistical significance levels are denoted as follows: *p-value  <  0.05; ** *p*-value  <  0.01; *** p-value <  0.001; **** *p*-value  <  0.0001, n. s.: not significant. Data are presented as mean ± s.e.m. or mean ± s.d. No statistical methods were used to predetermine sample size.

### Data reporting

No statistical methods were used to predetermine sample size. The experiments were not randomized, and the investigators were not blinded to allocation during experiments and outcome assessment.

### Reporting summary

Further information on research design is available in the Nature Portfolio Reporting Summary linked to this article.

### Supplementary information

Supplementary Information

Peer Review File

Description of Additional Supplementary Files

Supplementary Data 1

Supplementary Data 2

Supplementary Data 3

Supplementary Data 4

Supplementary Data 5

Supplementary Data 6

Supplementary Data 7

Supplementary Data 8

Reporting Summary

### Source data

Source Data

## Data Availability

The authors declare that the data supporting the findings of this study are available within the paper and its Supplementary Information, and available from the corresponding author upon request. All RNA-seq, ChIP-seq, and ATAC-seq data generated in this study have been deposited in the NCBI Gene Expression Omnibus (GEO) database and are accessible through the GEO SuperSeries accession number GSE193648. Source data are provided in this paper.
