# Supplementary material for: TAZ2 truncation confers overactivation of p300 and cellular vulnerability to HDAC inhibition
Source: Nat Commun. 2023 Sep 2;14:5362. doi: 10.1038/s41467-023-41245-2 (PMC10475075; doi:10.1038/s41467-023-41245-2)
Supplement: Supplementary file 1 — Supplementary Information [file 41467_2023_41245_MOESM1_ESM.pdf]

Supplementary Information for

TAZ2 truncation confers overactivation of p300 and cellular vulnerability to HDAC inhibition

Longxia Xu[1,3], Hongwen Xuan[1,3], Wei He[2], Liang Zhang[2], Mengying Huang[1], Kuai Li[1], Hong Wen[1], Han Xu[2], Xiaobing Shi[1,*]

[1]Department of Epigenetics, Van Andel Institute, Grand Rapids, MI, USA

[2]Department of Epigenetics and Molecular Carcinogenesis, The University of Texas MD Anderson Cancer Center, Houston, TX, USA

[3]These authors contributed equally: Longxia Xu, Hongwen Xuan.

*Correspondence: e-mail: Xiaobing.Shi@vai.org

This PDF file contains Supplementary Figures 1-9 and Supplementary Table 1.

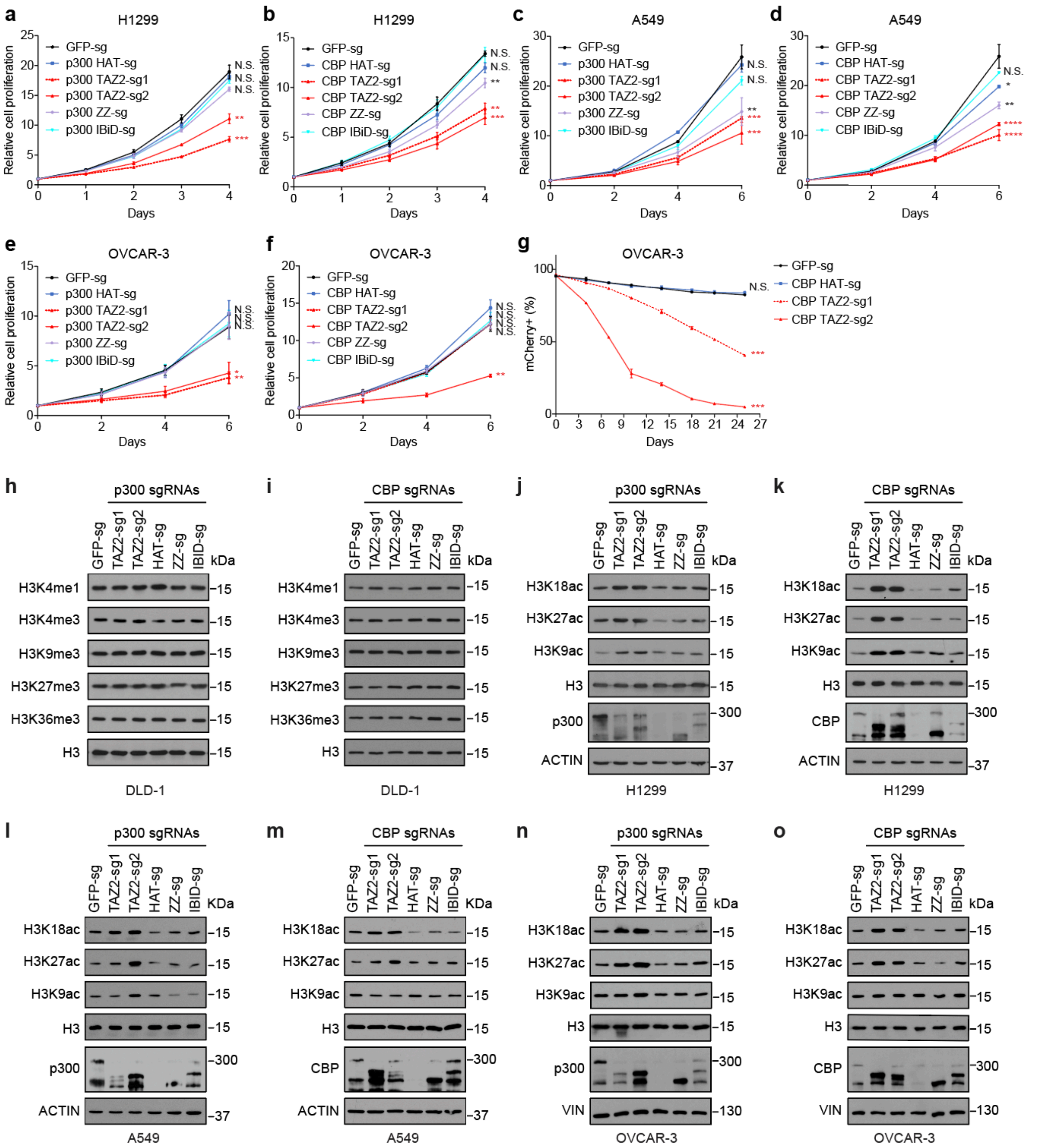

Supplementary Fig. 1. P300/CBP TAZ2 domain truncations inhibit cancer cell growth and increase histone H3K18 and H3K27 acetylation. a-f, Growth curves of H1299, A549 and OVCAR-3 cells stably expressing sgRNAs targeting the HAT, TAZ2, ZZ, and IBiD domains of p300 (**a-c**) or CBP (**d-f**). A GFP sgRNA was used as a control. Data are shown as mean ± s.e.m. of n=3, 4, 2, 2, 5, and 3 biological repeats, respectively. Compared to GFP-sg, p-values of HAT-sg, TAZ2-sg1, TAZ2-sg2, ZZ-sg and IBiD-sg in (**a**) are 0.4253, 0.0009, 0.0041, 0.0783 and 0.1872, respectively; p-values in (**b**) are 0.1765, 0.0012, 0.0007, 0.0061 and 0.9784, respectively; p-values in (**c**) are 0.86, 0.0002, 0.0003, 0.0086 and 0.0665, respectively; p-values in (**d**) are 0.0319, <0.0001, <0.0001, 0.0026 and 0.3598, respectively; p-values in (**e**) are 0.746, 0.0054, 0.0243, 0.9321 and 0.9184, respectively; p-values in (**f**) are 0.305, 0.919, 0.0029, 0.9529 and 0.9721, respectively. **g,** Competitive cell growth assay between OVCAR-3 cells expressing sgRNAs targeting CBP TAZ2 or HAT domains and control cells expressing a GFP-sg. sgRNA-expressing cells were monitored by the co-expressed mCherry and are shown as percentage of mCherry^{+} cells to the total cell populations. Data are representative of 2 biological experiments and presented as mean ± s.d.. Compared to GFP-sg, p-value=0.719 for CBP HAT-sg, p-value=0.0009 for CBP TAZ2-sg1; p-value=0.0001 for CBP TAZ2-sg2.In (**a-g**),

*, **, ***, **** indicate p-values <0.05, <0.01, <0.001, and <0.0001, respectively. N.S.: not significant (Two-way ANOVA). **h, i,** Western blots of histone methylation in DLD-1 cells expressing the indicated sgRNAs targeting individual domains of p300 (**h**) and CBP (**i**). **j-o,** Western blots of histone acetylation in H1299, A549 and OVCAR-3 cells expressing the indicated sgRNAs targeting individual domains of p300 (**j, l, n**) and CBP (**k, m, o**). Histone H3 and H3K9ac are shown for comparison. Actin and Vinculin were used as loading controls. In (**h**-**o**), all blots are representative of at least 2 biological experiments.

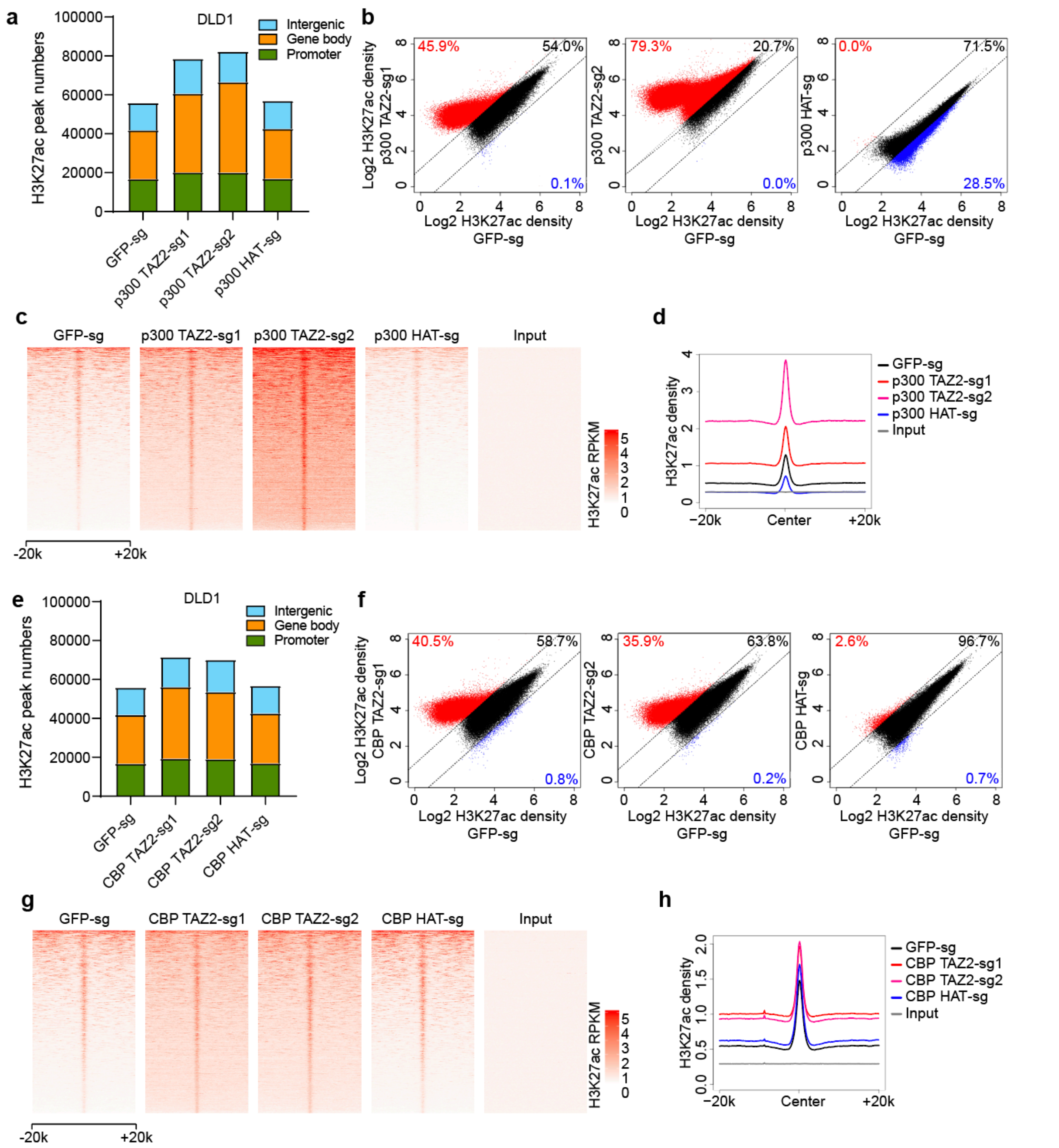

Supplementary Fig. 2. Genome-wide increase of H3K27ac in DLD-1 cells with p300/CBP TAZ2 domain truncations. **a,** Bar plot of H3K27ac ChIP-seq peak numbers in DLD-1 cells expressing p300 TAZ2 and HAT sgRNAs or the control GFP sgRNA (details in Supplementary Data 2). **b,** Dot plots of log_2 reads densities of H3K27ac ChIP-seq peaks from cells expressing p300 TAZ2 or HAT sgRNAs compared to the control GFP-sgRNA cells. Red and blue indicate relatively high and low H3K27ac signals (FC>1.5 and FDR<0.05), respectively. **c,** Heatmaps of H3K27ac ChIP-seq densities centered on H3K27ac peaks across a ± 20-kb window in cells as in (**a**). RPKM: Reads Per Kilobase Million. **d,** Average profiles of H3K27ac ChIP-seq densities centered on H3K27ac peaks in cells as in (**a**). **e,** Bar plots of H3K27ac ChIP-seq peak numbers in DLD-1 cells expressing CBP TAZ2 or HAT sgRNAs or the control GFP sgRNA (details in Supplementary Data 2). **f,** Dot plots of log_2 reads densities of H3K27ac ChIP-seq peaks from cells expressing CBP TAZ2 or HAT sgRNAs compared to the control GFP-sgRNA cells. Red and blue indicate relatively high and low H3K27ac signals (FC>1.5 and FDR<0.05), respectively. **g,** Heatmaps of H3K27ac ChIP-seq densities centered on H3K27ac peaks across a ± 20-kb window in cells as in (**e**). **h,** Average profiles of H3K27ac ChIP-seq densities centered on H3K27ac peaks in cells as in (**e**).

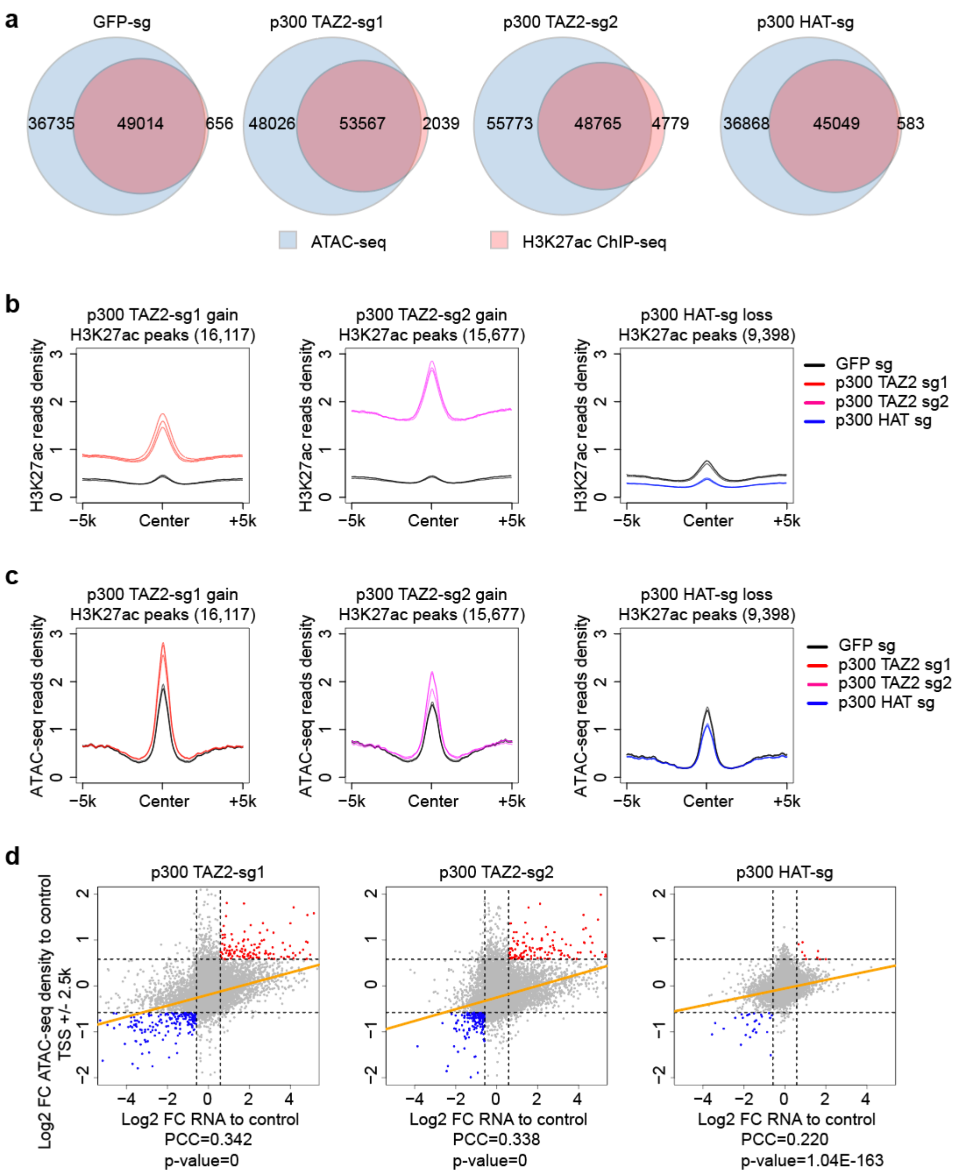

Supplementary Fig. 3. Elevated histone acetylation by p300 TAZ2 truncations increases chromatin accessibility. a, Venn diagrams of overlapping peaks between ATAC-seq peaks (light blue) and H3K27ac ChIP-seq peaks (light red) in OVCAR-3 cells expressing p300 TAZ2 or HAT sgRNAs or the control GFP sgRNA. ATAC-seq peaks and H3K27ac ChIP-seq peaks used for analysis were shared peaks of n=3 biological replicates in each group (details in Supplementary Data 2 and 3). *P*-values for all four intersections between ATAC-seq peaks and H3K27ac ChIP-seq peaks are <2e-4 (Permutation test). **b, c,** Average profiles of H3K27ac ChIP-seq densities (**b**) and ATAC-seq reads densities (**c**) centered on gained H3K27ac peaks in cells expressing p300 TAZ2 sgRNAs (left and middle panels) and lost peaks in cells expressing p300 HAT sgRNA (right panel) compared to cells expressing a control GFP sgRNA. Each line represents a biological replicate of n=3. **d**, Correlation between fold changes of promoter ATAC-seq density and gene expression in cells expressing p300 TAZ2 (left and middle) and HAT (right) sgRNAs compared to the control cells. Red and blue dots represent genes with increased or decreased accessibility and expression (FC>1.5), respectively. Orange lines indicate fitted linear models. PCC: Pearson correlation coefficient.

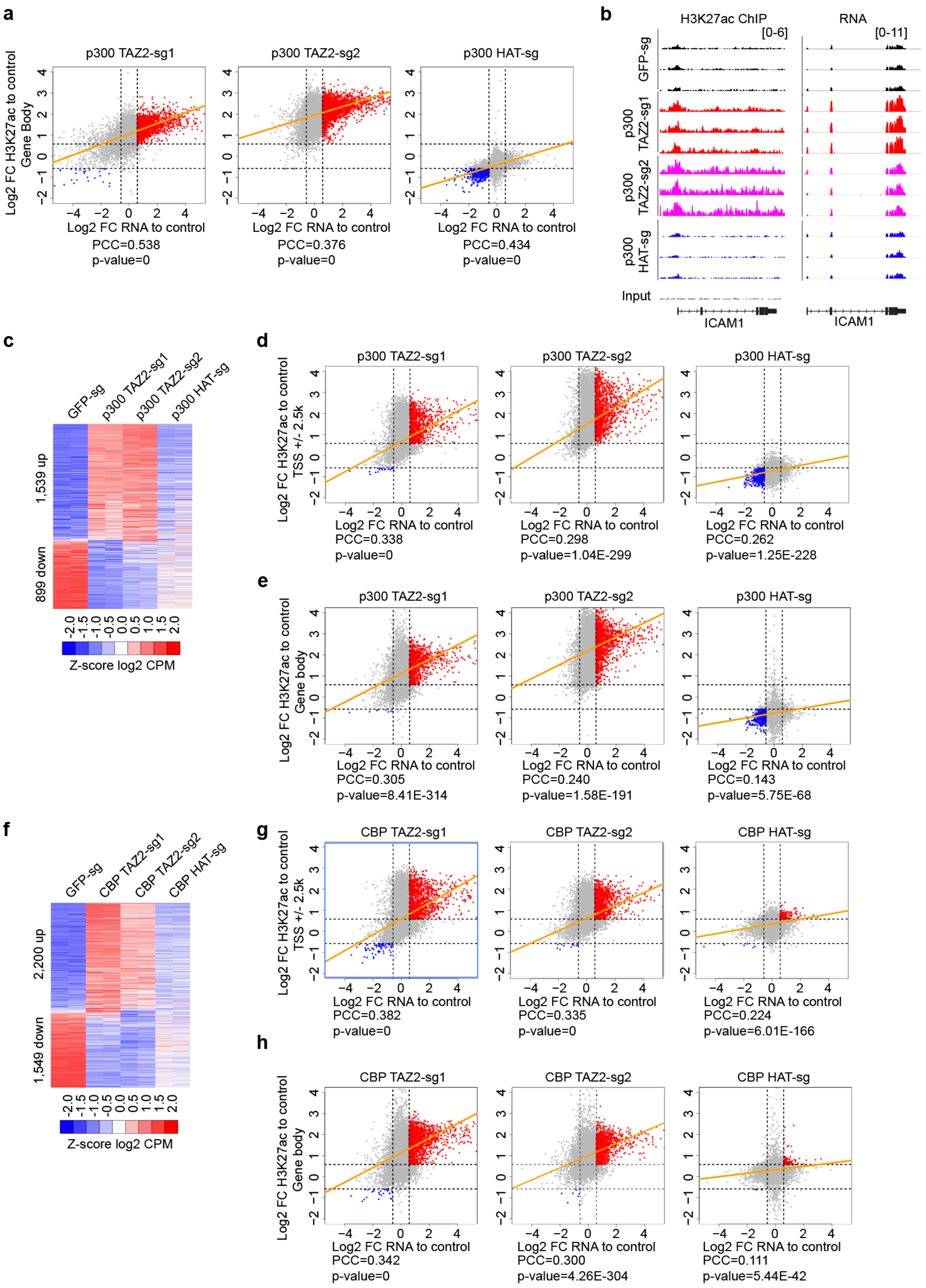
a
p300 TAZ2-sg1
p300 TAZ2-sg2
p300 HAT-sg
Log2 FC H3K27ac to control
Gene Body
Log2 FC RNA to control
PCC=0.538
p-value=0
PCC=0.376
p-value=0
PCC=0.434
p-value=0
b
H3K27ac ChIP
RNA
[0-6]
[0-11]
GFP-sg
p300 TAZ2-sg1
p300 TAZ2-sg2
p300 HAT-sg
Input
ICAM1
c
GFP-sg
p300 TAZ2-sg1
p300 TAZ2-sg2
p300 HAT-sg
1,539 up
899 down
-2.0 -1.5 -1.0 -0.5 0.0 0.5 1.0 1.5 2.0
Z-score log2 CPM
d
Log2 FC H3K27ac to control
TSS +/- 2.5k
PCC=0.338
p-value=0
PCC=0.298
p-value=1.04E-299
PCC=0.262
p-value=1.25E-228
e
Log2 FC H3K27ac to control
Gene body
PCC=0.305
p-value=8.41E-314
PCC=0.240
p-value=1.58E-191
PCC=0.143
p-value=5.75E-68
f
GFP-sg
CBP TAZ2-sg1
CBP TAZ2-sg2
CBP HAT-sg
2,200 up
1,549 down
-2.0 -1.5 -1.0 -0.5 0.0 0.5 1.0 1.5 2.0
Z-score log2 CPM
g
CBP TAZ2-sg1
CBP TAZ2-sg2
CBP HAT-sg
Log2 FC H3K27ac to control
TSS +/- 2.5k
PCC=0.382
p-value=0
PCC=0.335
p-value=0
PCC=0.224
p-value=6.01E-166
h
Log2 FC H3K27ac to control
Gene body
PCC=0.342
p-value=0
PCC=0.300
p-value=4.26E-304
PCC=0.111
p-value=5.44E-42

Supplementary Fig. 4. Increased histone acetylation by p300/CBP TAZ2 truncations is associated with enhanced gene expression in OVCAR-3 and DLD-1 cells. **a,** Correlation between fold changes of gene body H3K27ac and gene expression in OVCAR-3 cells expressing p300 TAZ2 (left and middle) and HAT (right) sgRNAs compared to the control cells. **b,** Integrative Genomics Viewer (IGV) views of H3K27ac ChIP-seq densities and RNA expression of *ICAM1* in OVCAR-3 cells expressing p300 TAZ2 or HAT sgRNAs or a control GFP sgRNA. Three biological replicates of each cell line are shown. **c,** Heatmap representation of genes differentially expressed in DLD-1 cells expressing p300 TAZ2 or HAT sgRNAs or a control GFP sgRNA. n=2 biological replicates. Red and blue indicate relatively high and low expression (FC>1.5 and FDR<0.05), respectively (details in Supplementary Data 4). CPM: counts per million. **d, e,** Correlation between gene expression and fold changes of H3K27ac at promoters (**d**) and gene bodies (**e**) in cells as in (**c**). **f,** Heatmap representation of genes differentially expressed in DLD-1 cells expressing CBP TAZ2 or HAT sgRNAs or a control GFP sgRNA. n=2 biological replicates. Red and blue indicate relatively high and low expression (FC>1.5 and FDR<0.05), respectively (details in Supplementary Data 4). **g, h,** Correlation between gene expression and fold changes of H3K27ac at promoters (**g**) and gene bodies (**h**) in cells as in (**f**). In (**a, d, e, g, h**), X-axis is $\log_2$ fold change of RNA expression and Y-axis is $\log_2$ fold change of H3K27ac levels. Red and blue dots represent genes with increased or decreased H3K27ac and gene expression (FC>1.5), respectively. Orange lines indicate fitted linear models. PCC: Pearson correlation coefficient.

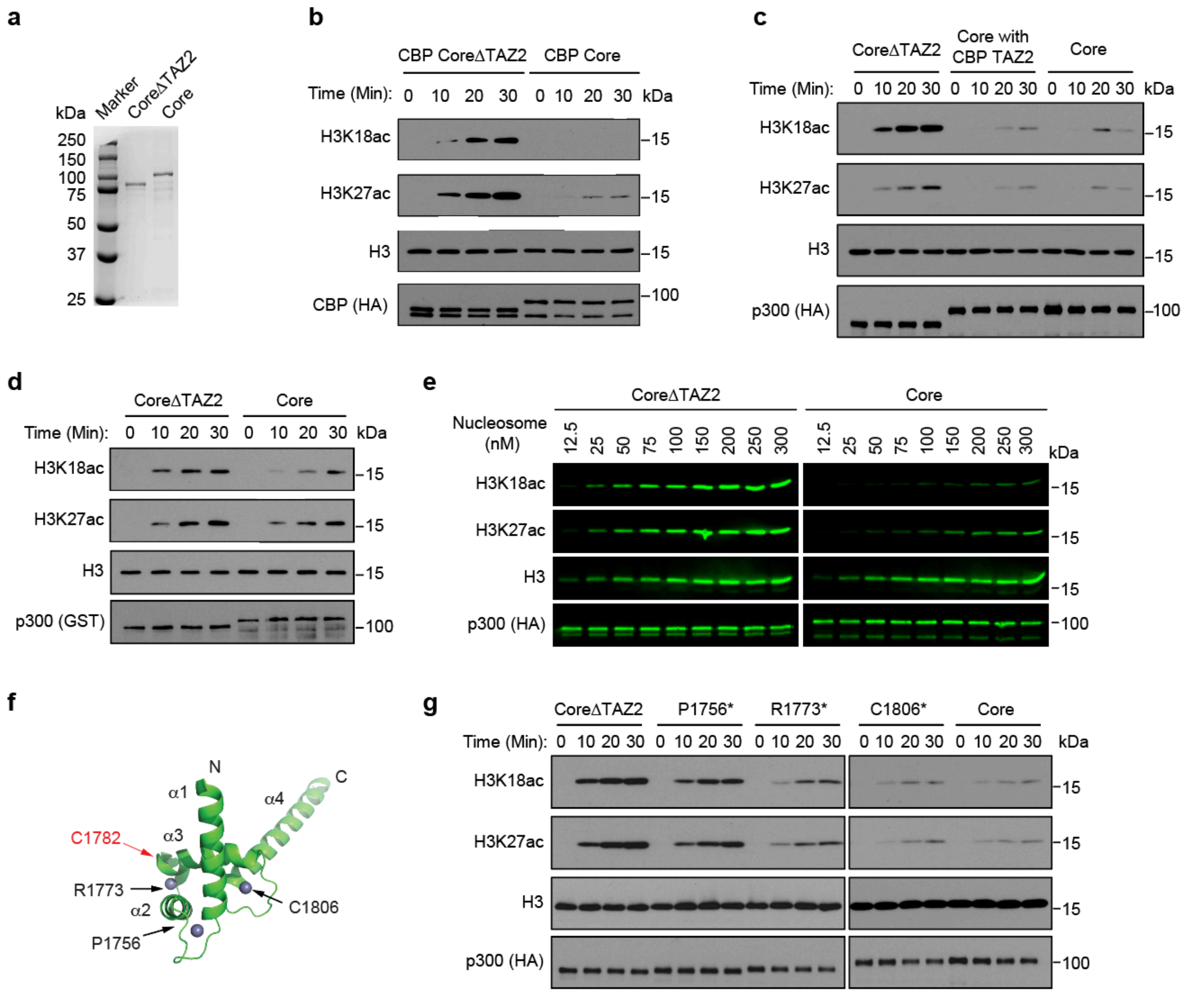

Supplementary Fig. 5. The TAZ2 domain inhibits p300/CBP HAT activity. **a,** Coomassie staining of purified p300 Core and CoreΔTAZ2 proteins from 293T cells. **b,** Western blots of *in vitro* HAT assays using the CBP Core and CoreΔTAZ2 proteins purified from 293T cells. **c,** Western blots of *in vitro* HAT assays using purified p300 Core, CoreΔTAZ2, and p300 Core with the swapped CBP TAZ2 domain. **d,** Western blots of *in vitro* HAT assays using recombinant GST-tagged p300 Core and CoreΔTAZ2 fragments purified from *E.coli*. **e,** Representative of quantitative fluorescent Western blots of HAT assays using the p300 Core and CoreΔTAZ2 and recombinant nucleosome for kinetic measurement. **f,** Crystal structure of p300 TAZ2 domain in cartoon (PDB: 3io2). The four alpha helices (α1-α4) are indicated. Red and black arrows indicate the amino acid positions for alpha helix truncations used in Figure **3e** and (**g**), respectively. **g,** Western blots of *in vitro* HAT assays using p300 Core, CoreΔTAZ2, and the indicated TAZ2 alpha helix truncation mutants. All blots are representative of 2 (**b, c**) and 3 (**a, d, e, g**) biological experiments.

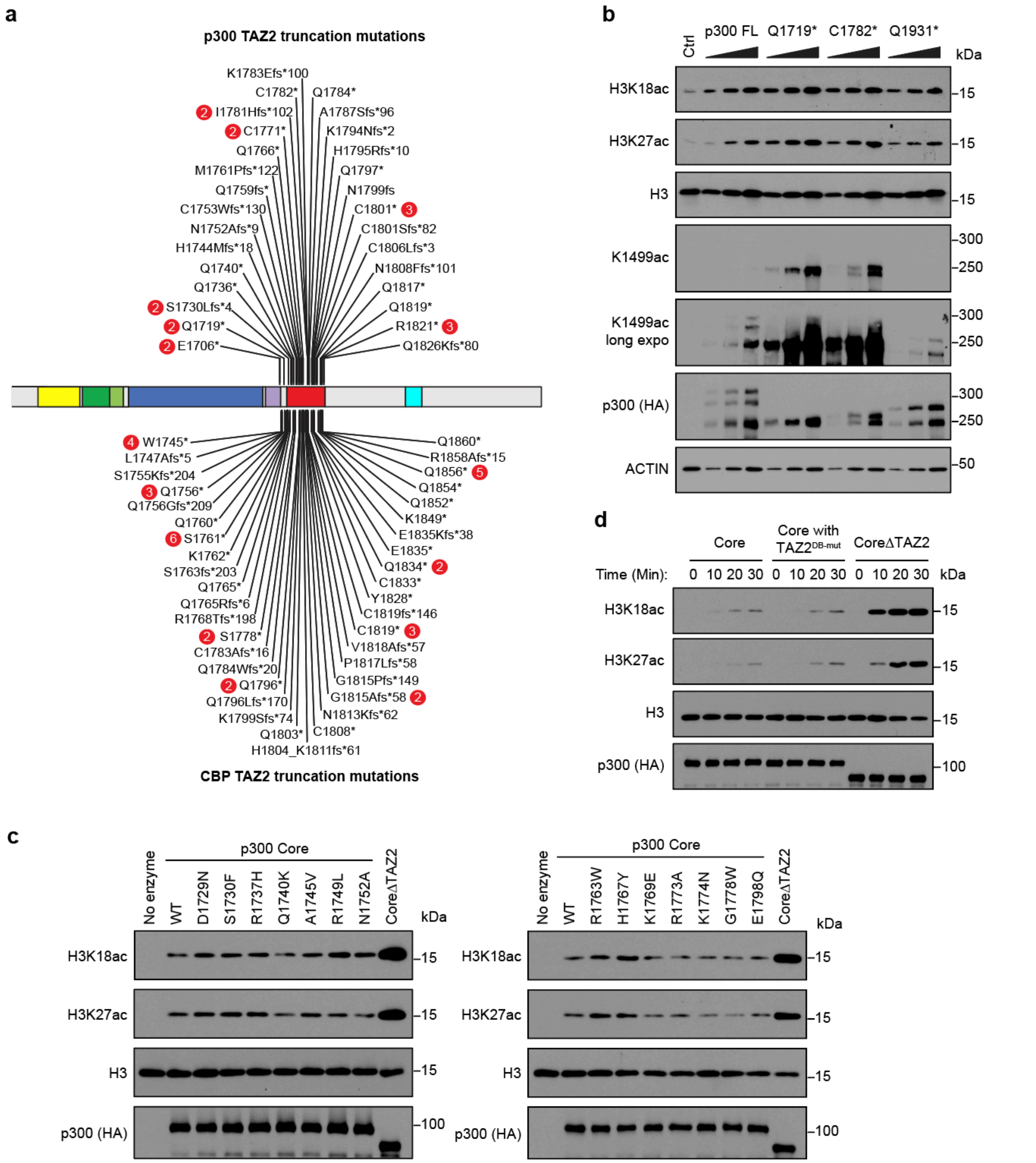

Supplementary Fig. 6. P300/CBP TAZ2 mutations in human cancers. **a,** Frameshift (fs) and nonsense mutations of *p300* and *CBP* identified in human cancer patients that lead to TAZ2 truncations. Data from COSMIC (https://cancer.sanger.ac.uk/cosmic). Numbers in red indicate number of cases reported. **b**, Western blots of whole cell extract from cells expressing full-length p300 and the indicated TAZ2 cancer mutants. TAZ2 truncation mutants showed higher p300 autoacetylation levels. **c**, Western blots of *in vitro* HAT assays using the purified p300 Core, CoreΔTAZ2, and p300 Core containing TAZ2 missense mutations found in cancer patients. **d**, Western blots of *in vitro* HAT assays using the purified p300 Core, CoreΔTAZ2, and p300 Core containing the DNA-binding deficient mutant TAZ2 (TAZ2$^{DB\text{-}mut}$: R1731E/K1794E/K1812E). All blots are representative data of 2 biological experiments.

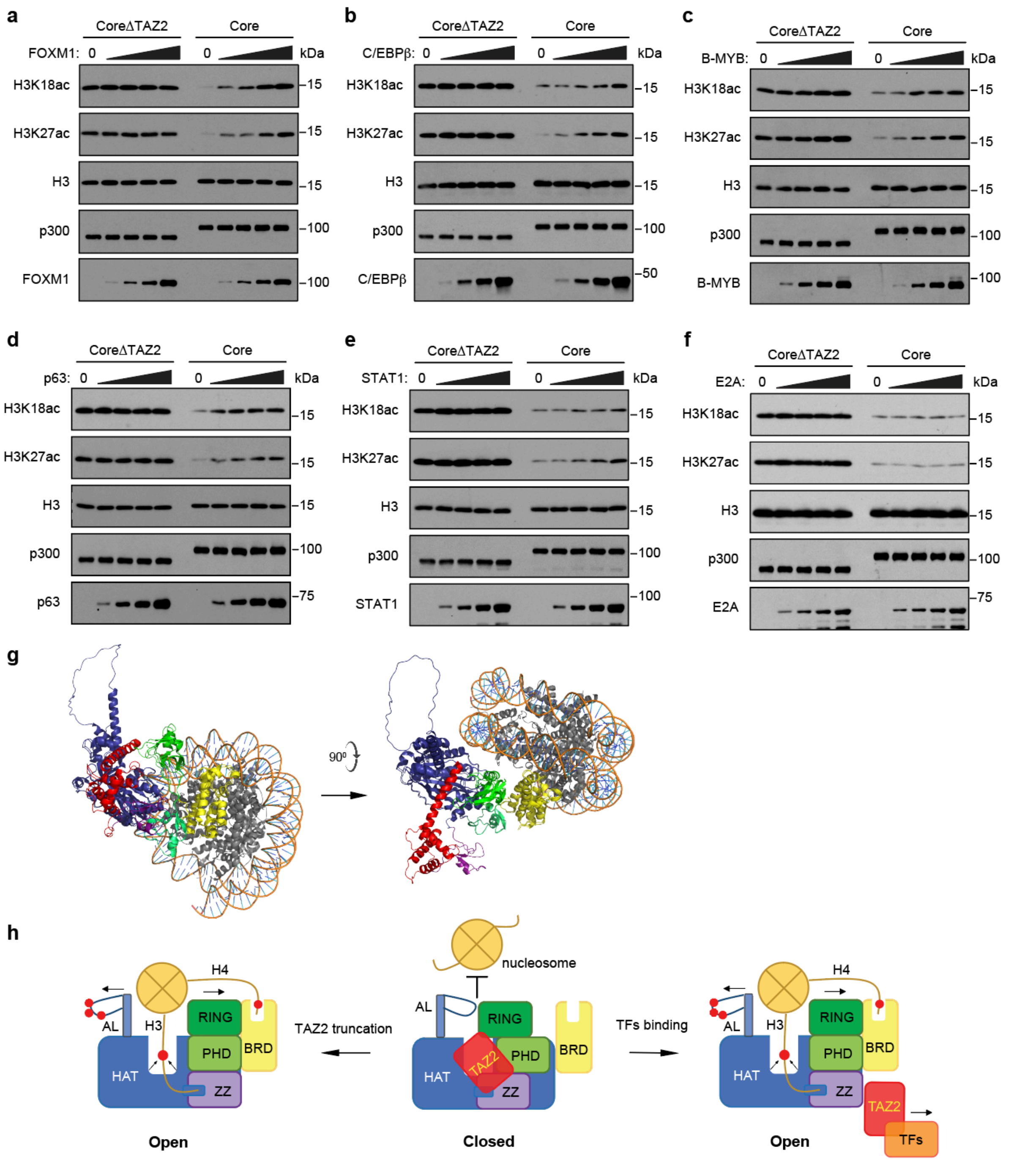

Supplementary Fig. 7. Transcription factors bind to TAZ2 and relieve TAZ2-mediated inhibition of p300 HAT activity. **a-f,** Western blots of *in vitro* HAT assays of p300 Core and CoreΔTAZ2 in the presence of increasing amounts of the indicated transcriptional factors. All blots are representative of at least 2 biological experiments. **g,** Superimposition of the structure of p300 Core (aa1048-1836) predicted by AlphaFold (https://alphafold.ebi.ac.uk/entry/Q09472) and the cryo-EM structure of p300 CoreΔTAZ2 (aa1035-1720) bound to nucleosome (PDB: 7w9v). The BRD, RING, PHD, HAT, ZZ and TAZ2 domains are shown in yellow, green, limegreen, blue, purple, and red, respectively. Histones are shown in dark gray and DNA in orange. **h,** Model for TAZ2-mediated inhibition of p300 HAT activity. In the closed state (middle), TAZ2 interacts with HAT, PHD and BRD, together with unacetylated AL (autoregulatory loop) and RING blocking the HAT active site, thus keeping HAT inactive. Upon TAZ2 truncation or TF binding that dissociates TAZ2 from other domains, hyperacetylated AL and RING move away from the active site, leaving the HAT active site open to substrate. In addition, binding of acetylated H4 to BRD and H3 to ZZ further promote the access of HAT to the histone substrate. Red dots: acetylation.

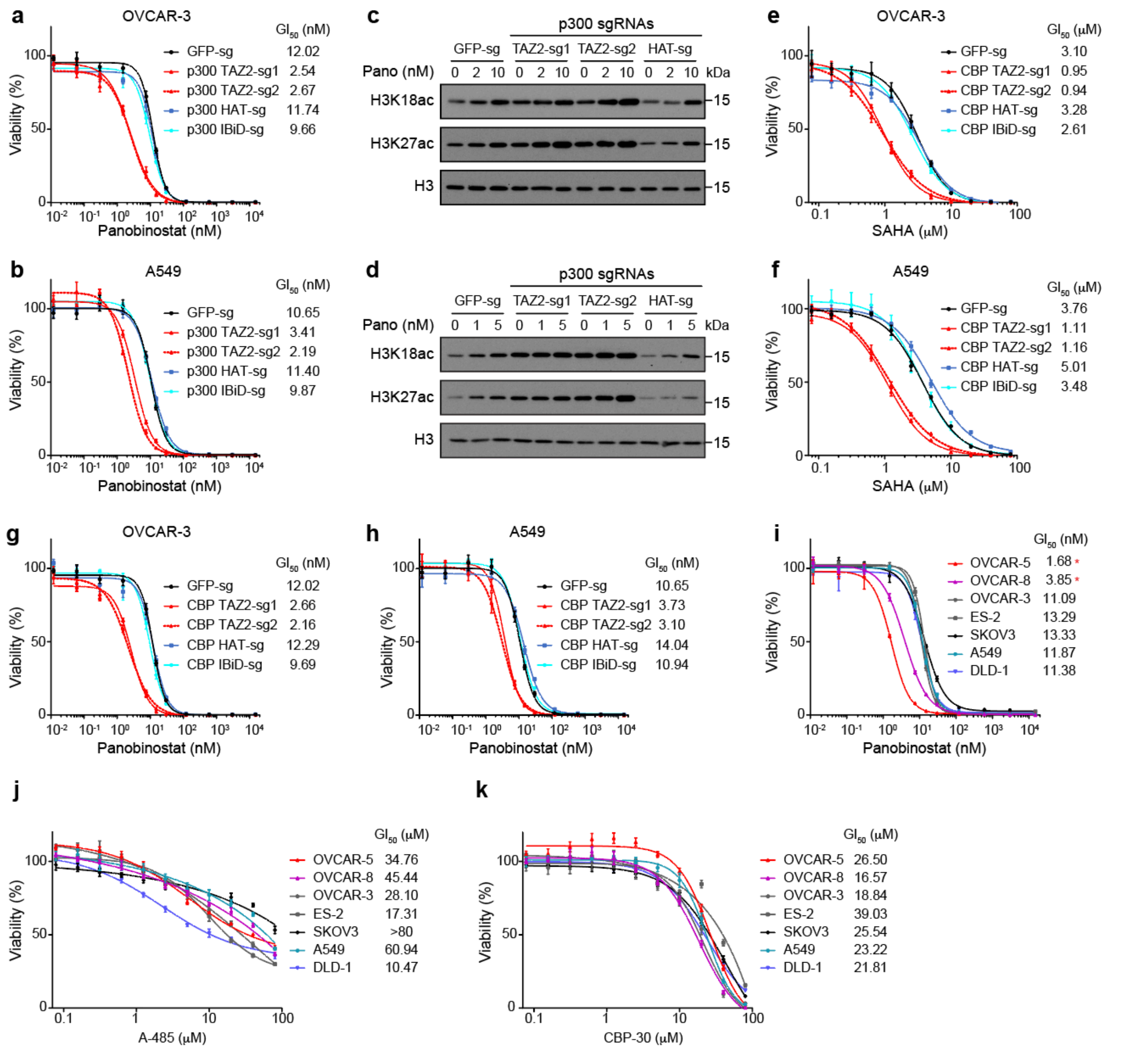

Supplementary Fig. 8. P300/CBP TAZ2 truncations sensitize cells to HDAC inhibitors. a, b, Cells with p300 TAZ2 truncation are sensitive to Panobinostat. Cell viability was measured for OVCAR-3 (**a**) and A549 (**b**) cells expressing p300 TAZ2 and HAT sgRNAs or a control GFP sgRNA and treated with different doses of Panobinostat for 3 days. **c, d,** Western blots showing Panobinostat-induced histone acetylation changes in cells as in (**a**, **b**). Blots are representative of 2 biological repeats. **e-h,** CBP TAZ2 truncated cells are sensitive to HDAC inhibitors. Cell viability was measured for OVCAR-3 and A549 cells expressing CBP TAZ2 and HAT sgRNAs or a control GFP sgRNA and treated with different doses of SAHA (**e,f**) or Panobinostat (**g,h**). **i-k,** Cancer cell lines with p300 TAZ2 truncations are sensitive to Panobinostat, but not to the p300/CBP inhibitors A-485 or CBP-30. Cell viability measurement for p300/CBP WT cell lines and the two cell lines containing p300 TAZ2 truncation mutations (OVCAR-5 and OVCAR-8, indicated by asterisks) treated with different doses of Panobinostat (**i**), A-485 (**j**) and CBP-30 (**k**). In (**a**, **b**, **e-k**), Data are representative of 3 biological repeats and presented as mean ± s.d.. GI_{50}, the drug concentration for 50% of maximal inhibition of cell proliferation.

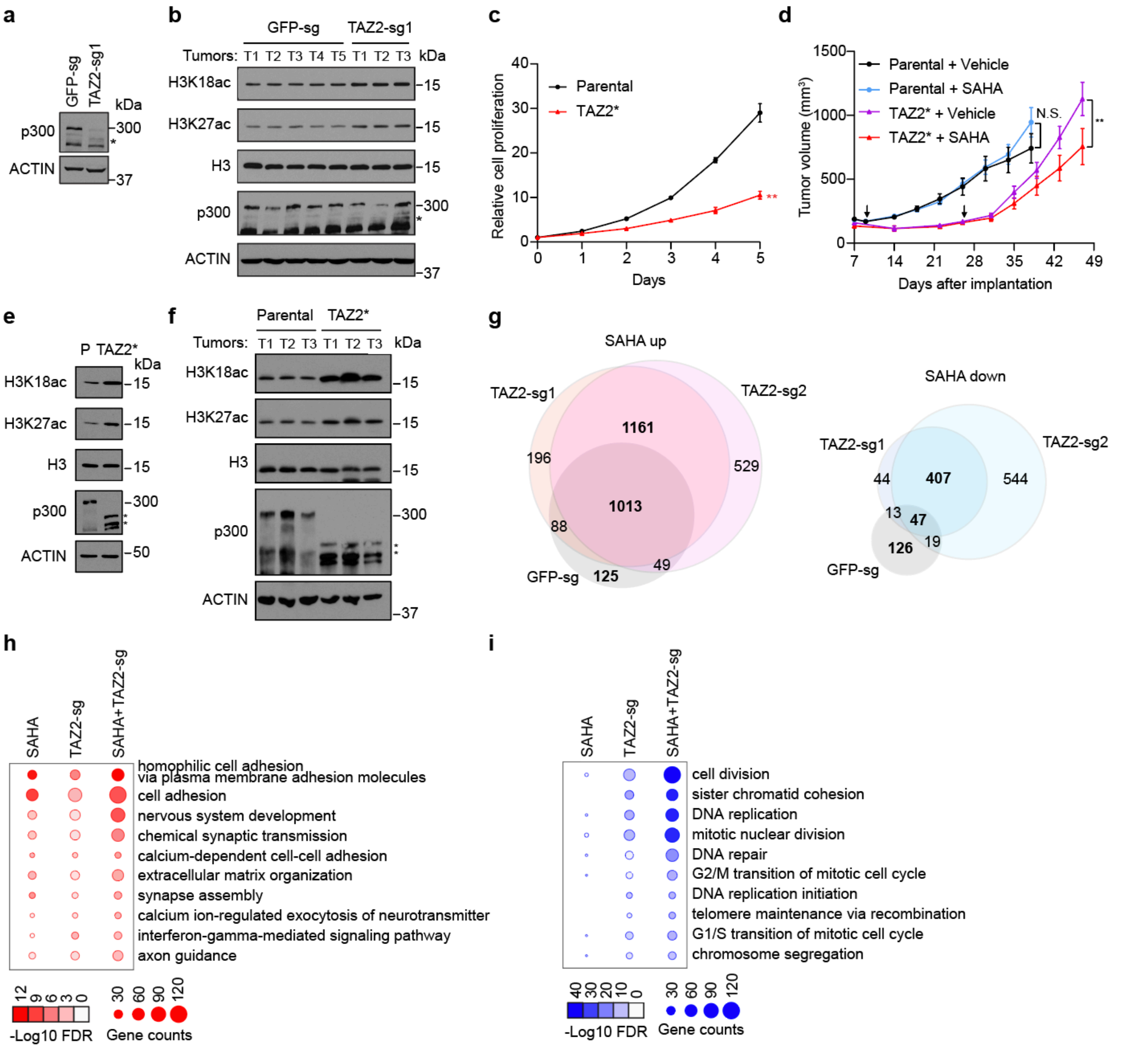

Supplementary Fig. 9. SAHA and p300 TAZ2 truncation synergize in gene induction. **a,b,** Western blots of A549 cells expressing p300 TAZ2-sg1 and GFP-sg before transplantation (**a**) and from xenografted tumors collected at the endpoint of SAHA treatment (**b**). Asterisks indicate TAZ2 truncated p300. All blots are representative data of 2 biological experiments. **c,** *In vitro* cell growth curves of the parental A549 and isogenic cells containing p300 TAZ2 truncation (TAZ2*). Error bars represent s.e.m. of 2 biological replicates. **p= 0.0015. **d,** Tumor growth of xenografted parental A549 cells and TAZ2* cells under vehicle and SAHA treatment (25mg/kg/day, daily IP). Arrows indicate the dates that treatment started. Data presented are mean ± s.e.m. of n=8, 9, 10, and 10 mice (from top to bottom). **p=0.0021. N.S.: not significant, p=0.2893(Two-way ANOVA). **e**,**f**, Western blots of the parental A549 cells (P) and TAZ2* before transplantation (**e**) and from the xenografted tumors collected at the endpoint of SAHA treatment (**f**). Asterisks indicate TAZ2 truncated p300 proteins. All blots are representative data of 2 biological experiments. **g**, Venn diagrams of the overlapping up (left panel) and downregulated (right panel) genes induced by SAHA treatment (0.5 μM, 24h) in A549 cells expressing p300 TAZ2 sgRNAs or a control GFP sgRNA. *P*-values for all pairs in both up and down regulated genes are < 2.2e-16 (Fisher's exact test). Genes presented in Figure **4g, h** are in bold and the full lists of genes are provided in Supplementary Data 7. **h, i**, Bubble plots of DAVID Gene Ontology biological processes enriched in up regulated genes (**h**) and down regulated genes (**i**) induced by SAHA treatment, p300 TAZ2 sgRNAs, and both (SAHA+TAZ2 sgRNAs) in A549 cells. The bubble size indicates the number of enriched genes, and the color density indicates -$\log_{10}$ FDR value of the terms. Red and blue colors indicate up and down regulated genes, respectively. More details are provided in Supplementary Data 8.

Supplementary Table 1. Antibodies used in this study

Antibodies	Source	Catalog #	RRID	Dilutions
Mouse monoclonal anti-H3K9ac	Active Motif	61251	AB_2793569	1:1000
Rabbit monoclonal anti-H3K9ac	Abcam	ab32129	AB_732920	1:1000
Rabbit monoclonal anti-H3K14ac	Abcam	ab52946	AB_880442	1:1000
Rabbit polyclonal anti-H3K18ac	Active Motif	39755	AB_2714186	1:1000
Rabbit monoclonal anti-H3K27ac	Abcam	ab177178	AB_2828007	1:1000
Rabbit polyclonal anti-H3K27ac	Abcam	ab4729	AB_2118291	1:1000
Rabbit polyclonal anti-H3K4me1	Abcam	ab8895	AB_306847	1:1000
Rabbit polyclonal anti-H3K4me3	Abcam	ab8580	AB_306649	1:1000
Rabbit polyclonal anti-H3K9me3	Abcam	ab8898	AB_306848	1:1000
Rabbit polyclonal anti-H3K27me3	Millipore	07-449	AB_310624	1:2000
Rabbit polyclonal anti-H3K36me3	Abcam	ab9050	AB_306966	1:1000
Rabbit polyclonal anti-H3	Abcam	ab1791	AB_302613	1:10000
Rabbit monoclonal anti-p300	CST	54062S	AB_2799450	1:1000
Rabbit monoclonal anti-CREBBP	CST	7389S	AB_2616020	1:1000
Mouse monoclonal anti-beta-Actin	Sigma	A1978	AB_476692	1:5000
Mouse monoclonal anti-vinculin	Santa Cruz	sc-25336	AB_628438	1:1000
Rabbit monoclonal anti-HA tag	CST	3724S	AB_1549585	1:5000
Mouse monoclonal anti-His tag	ZSGB-Bio	TA-02	AB_2801388	1:5000
Rabbit polyclonal anti-GST	Santa Cruz	sc-459	AB_631586	1:1000
Rabbit polyclonal anti-Acetyl-p300 (K1499)	CST	4771S	AB_2262406	1:2000
Mouse monoclonal anti-p53	Millipore	OP43	AB_213402	1:1000
Rabbit monoclonal anti-GFP	CST	2956S	AB_1196615	1:1000
Rabbit polyclonal anti-PHF6	Sigma	HPA001023	AB_1079606	1:1000
Rabbit polyclonal anti-FBW7	Bethyl	A301-720A	AB_1210897	1:1000
Rabbit polyclonal anti-Acetylated-Lysine	CST	9441S	AB_331805	1:2000
Mouse monoclonal anti-beta-Tubulin	Sigma	T8328	AB_1844090	1:5000
IRDye® 800CW Goat anti-Rabbit IgG	LI-COR	926-32211	AB_621843	1:5000
Peroxidase-AffiniPure Goat Anti-Rabbit IgG	Jackson Labs	111-035-114	AB_2307391	1:20000
Peroxidase-AffiniPure Goat Anti-Mouse IgG	Jackson Labs	115-035-003	AB_10015289	1:20000
