# Supplementary material for: TAZ2 truncation confers overactivation of p300 and cellular vulnerability to HDAC inhibition
Source: Nat Commun. 2023 Sep 2;14:5362. doi: 10.1038/s41467-023-41245-2 (PMC10475075; doi:10.1038/s41467-023-41245-2)
Supplement: Supplementary file 3 — Description of Additional Supplementary Files [file 41467_2023_41245_MOESM3_ESM.pdf]

Description of Additional Supplementary Files

File Name: Supplementary Data 1
Description: Sanger sequencing of p300/CBP TAZ2 and ZZ sgRNAs targeted loci.

File Name: Supplementary Data 2
Description: MACS2 called H3K27ac ChIP-seq peaks in OVCAR-3 and DLD-1 cells.

File Name: Supplementary Data 3
Description: MACS2 Called ATAC-seq peaks in OVCAR3.

File Name: Supplementary Data 4
Description: edgeR generated CPM and log2 FC of RNA-seq in OVCAR3 and DLD-1.

File Name: Supplementary Data 5
Description: Enriched Gene Ontology Biological Processes terms in OVCAR3 and DLD-1 from DAVID Go analysis.

File Name: Supplementary Data 6
Description: Crosslinked peptides of the p300 core identified by cross-linking mass spectrometry (CLMS).

File Name: Supplementary Data 7
Description: edgeR generated log2 FC and log2 CPM of RNA-seq in A549.

File Name: Supplementary Data 8
Description: Enriched Gene Ontology Biological Processes terms in A549 from DAVID Go analysis.
