# Supplementary material for: TAZ2 truncation confers overactivation of p300 and cellular vulnerability to HDAC inhibition
Source: Nat Commun. 2023 Sep 2;14:5362. doi: 10.1038/s41467-023-41245-2 (PMC10475075; doi:10.1038/s41467-023-41245-2)
Supplement: Supplementary file 12 — Reporting Summary [file 41467_2023_41245_MOESM12_ESM.pdf]

nature portfolio

Corresponding author(s): Xiaobing Shi

Last updated by author(s): Aug 8, 2023

Reporting Summary

Nature Portfolio wishes to improve the reproducibility of the work that we publish. This form provides structure for consistency and transparency in reporting. For further information on Nature Portfolio policies, see our Editorial Policies and the Editorial Policy Checklist.

Statistics

For all statistical analyses, confirm that the following items are present in the figure legend, table legend, main text, or Methods section.

n/a	Confirmed	
☐	☒	The exact sample size (*n*) for each experimental group/condition, given as a discrete number and unit of measurement
☐	☒	A statement on whether measurements were taken from distinct samples or whether the same sample was measured repeatedly
☐	☒	The statistical test(s) used AND whether they are one- or two-sided<br>*Only common tests should be described solely by name; describe more complex techniques in the Methods section.*
☐	☒	A description of all covariates tested
☐	☒	A description of any assumptions or corrections, such as tests of normality and adjustment for multiple comparisons
☐	☒	A full description of the statistical parameters including central tendency (e.g. means) or other basic estimates (e.g. regression coefficient) AND variation (e.g. standard deviation) or associated estimates of uncertainty (e.g. confidence intervals)
☐	☒	For null hypothesis testing, the test statistic (e.g. *F*, *t*, *r*) with confidence intervals, effect sizes, degrees of freedom and *P* value noted<br>*Give P values as exact values whenever suitable.*
☒	☐	For Bayesian analysis, information on the choice of priors and Markov chain Monte Carlo settings
☒	☐	For hierarchical and complex designs, identification of the appropriate level for tests and full reporting of outcomes
☐	☒	Estimates of effect sizes (e.g. Cohen's *d*, Pearson's *r*), indicating how they were calculated

Our web collection on statistics for biologists contains articles on many of the points above.

Software and code

Policy information about availability of computer code

Data collection	Biotek Synergy HT microplate reader was used to collect CellTiter-Glo luminescent data; ChemiDoc MP Imaging System was use to collect fluorescent signal for quantitative Western blots; Orbitrap Fusion Lumos (Thermo Scientific)<br>coupled on-line with an Ultimate 3000 HPLC (Thermo Scientific) were used for LC-MS analysis of cross-linked peptides
Data analysis	GraphPad Prism (v9) for cell and tumor growth analysis and statistics; Image lab (v6.0.1) for fluorescent western blot analysis; ProteoWizard MSConvert (v. 3.0.10738) and Protein Prospector (v.5.19.1, University of California, San Francisco) for mass spectrometry data analysis; ProTiler (v1.0) for tiling-sgRNA screen analysis; HISAT2 (v2.1.0), MACS2 (v2.1.2), bedtools (v2.27.1), Danpos2 (v2.2.2), and R (v3.5.1) for ChIP-seq analysis; HISAT2 (v2.1.0), HTSeq (v0.11.3), edgeR (v3.16.5), DAVID 6.8, Danpos2 (v2.2.2), and R (v3.5.1) for RNA-seq analysis; HISAT2 (v2.1.0), Samtools (v1.9), MACS2 (v2.1.2), bedtools (v2.27.1), and Danpos2 (v2.2.2) for ATAC-seq analysis.

For manuscripts utilizing custom algorithms or software that are central to the research but not yet described in published literature, software must be made available to editors and reviewers. We strongly encourage code deposition in a community repository (e.g. GitHub). See the Nature Portfolio guidelines for submitting code & software for further information.

Data

Policy information about availability of data

All manuscripts must include a data availability statement. This statement should provide the following information, where applicable:

- Accession codes, unique identifiers, or web links for publicly available datasets
- A description of any restrictions on data availability
- For clinical datasets or third party data, please ensure that the statement adheres to our policy

The authors declare that the data supporting the findings of this study are available within the paper and its Supplementary Information. All RNA-seq, ChIP-seq, and ATAC-seq data described in the manuscript have been deposited in the NCBI Gene Expression Omnibus (GEO) database (GSE193648). Publicly available databases used in this study are available at the indicated locations: https://depmap.org/portal/download/api/download?file_name=ccle%2Fccle_2019%2FCCLE_GlobalChromatinProfiling_20181130.csv&bucket=depmap-external-downloads
p300/CBP TAZ2 mutations in human cancer were downloaded from COSMIC (https://cancer.sanger.ac.uk/cosmic)
hg38 genome sequence and annotation were downloaded from GENCODE (https://www.gencodegenes.org/human/)

Research involving human participants, their data, or biological material

Policy information about studies with human participants or human data. See also policy information about sex, gender (identity/presentation), and sexual orientation and race, ethnicity and racism.

Reporting on sex and gender	*Use the terms sex (biological attribute) and gender (shaped by social and cultural circumstances) carefully in order to avoid confusing both terms. Indicate if findings apply to only one sex or gender; describe whether sex and gender were considered in study design; whether sex and/or gender was determined based on self-reporting or assigned and methods used. Provide in the source data disaggregated sex and gender data, where this information has been collected, and if consent has been obtained for sharing of individual-level data; provide overall numbers in this Reporting Summary. Please state if this information has not been collected. Report sex- and gender-based analyses where performed, justify reasons for lack of sex- and gender-based analysis.*
Reporting on race, ethnicity, or other socially relevant groupings	*Please specify the socially constructed or socially relevant categorization variable(s) used in your manuscript and explain why they were used. Please note that such variables should not be used as proxies for other socially constructed/relevant variables (for example, race or ethnicity should not be used as a proxy for socioeconomic status). Provide clear definitions of the relevant terms used, how they were provided (by the participants/respondents, the researchers, or third parties), and the method(s) used to classify people into the different categories (e.g. self-report, census or administrative data, social media data, etc.) Please provide details about how you controlled for confounding variables in your analyses.*
Population characteristics	*Describe the covariate-relevant population characteristics of the human research participants (e.g. age, genotypic information, past and current diagnosis and treatment categories). If you filled out the behavioural & social sciences study design questions and have nothing to add here, write "See above."*
Recruitment	*Describe how participants were recruited. Outline any potential self-selection bias or other biases that may be present and how these are likely to impact results.*
Ethics oversight	*Identify the organization(s) that approved the study protocol.*

Note that full information on the approval of the study protocol must also be provided in the manuscript.

Field-specific reporting

Please select the one below that is the best fit for your research. If you are not sure, read the appropriate sections before making your selection.

☒ Life sciences ☐ Behavioural & social sciences ☐ Ecological, evolutionary & environmental sciences

For a reference copy of the document with all sections, see nature.com/documents/nr-reporting-summary-flat.pdf

Life sciences study design

All studies must disclose on these points even when the disclosure is negative.

Sample size	For in vitro experiments, at least three biologically independent experiments were performed for all experiments unless otherwise stated. No statistical method was used to predetermine sample size. Such sample sizes are typical for the in vitro experiments and sufficient for a statistical analysis. For in vivo experiments, a sample size of n = 8-10 mice per group were used, which is sufficient to generate statistically significant results. No statistical method was used to predetermine sample size.
Data exclusions	No data exclusions occurred in this study.
Replication	The replication numbers were described in the corresponding figure legends.

Randomization

For in vitro experiments, cells were randomly allocated into control and experimental groups. For in vivo experiments, mice were randomly assigned to experimental and control groups when tumor size reached ~150mm3.

Blinding

The investigators were not blinded to group allocation during data collection or analysis because experimental results are quantitative in nature, not readily subject to investigator bias. To ensure consistent experimental conditions, all control and experimental samples were processed in parallel.

Reporting for specific materials, systems and methods

We require information from authors about some types of materials, experimental systems and methods used in many studies. Here, indicate whether each material, system or method listed is relevant to your study. If you are not sure if a list item applies to your research, read the appropriate section before selecting a response.

Materials & experimental systems

n/a	Involved in the study	
☐	☒	Antibodies
☐	☒	Eukaryotic cell lines
☒	☐	Palaeontology and archaeology
☐	☒	Animals and other organisms
☒	☐	Clinical data
☒	☐	Dual use research of concern
☒	☐	Plants

Methods

n/a	Involved in the study	
☐	☒	ChIP-seq
☒	☐	Flow cytometry
☒	☐	MRI-based neuroimaging

Antibodies

Antibodies used

Target,Supplier,Catalog No., clone name, lot number, Application/ Dilution or amount
Rabbit monoclonal anti-H3K9ac, abcam, ab32129, Y28, WB/1:1000
Rabbit monoclonal anti-H3K14ac, abcam, ab52946, EP964Y, GR1497415, WB/1:1000
Rabbit monoclonal anti-H3K27ac, abcam, ab177178, EP16602, GR3202987-21, WB/1:1000
Rabbit polyclonal anti-H3K27ac, abcam, ab4729, GR306603-1, WB/1:1000
Rabbit polyclonal anti-H3K4me1, abcam, ab8895, WB/1:1000
Rabbit polyclonal anti-H3K4me3, abcam, ab8580, GR190237-1, WB/1:1000
Rabbit polyclonal anti-H3K9me3, abcam, ab8898, GR39149-1, WB/1:1000
Rabbit polyclonal anti-H3K36me3, abcam, ab9050, GR27079-1), WB/1:1000
Mouse monoclonal anti-H3K9ac, ActiveMotif, 61251, 1B10, 08216003, WB/1:1000
Rabbit polyclonal anti-H3K18ac, ActiveMotif, 39755, 06710001, WB/1:1000
Rabbit polyclonal anti-H3K27me3, Millipore, 07-449, 2826067, WB/1:2000
Rabbit polyclonal anti-H3, Abcam, ab1791, GR135489-1,WB/1:10000
Rabbit monoclonal anti-p300, Cell Signaling Technology, 54062S, D2X6N, lot 1, WB/1:1000
Rabbit monoclonal anti-CBP, Cell Signaling Technology, 7389S, D6C5, lot 5, WB/1:1000
Rabbit monoclonal anti-HA-Tag, Cell Signaling Technology, 3724S, C29F4, Lot 8, WB/1:5000
Rabbit polyclonal anti-Acetyl-CBP (Lys1535)/p300 (Lys1499), Cell Signaling Technology, 4771S, lot 3, WB/1:2000
Mouse monoclonal anti-beta-Actin, Sigma, A1978, AC-15, 043M4840V, WB/1:5000
Mouse monoclonal anti-vinculin, Santa Cruz, sc-25336, H-10, F0619, WB/1:1000
Rabbit polyclonal anti-GST , Santa Cruz, sc-459, K0713, WB/1:1000
Mouse monoclonal anti-His tag , ZSGB-Bio, TA-02, OTI2B5, WB/1:5000
IRDye® 800CW Goat anti-Rabbit IgG, LI-COR, 926-32211, WB/1:5000
HRP-conjugated Goat anti-rabbit IgG, Jackson ImmunoResearch, 111-035-114, WB/1:20000
HRP-conjugated Goat anti-mouse IgG, Jackson ImmunoResearch, 115-035-003, WB/1:20000
Rabbit monoclonal anti-H3K27ac, abcam, ab177178, EP16602, GR3202987-21, ChIP/1.5ul

Validation

All these antibodies were commercially obtained and validated by vendors and multiple published studies, see manufacture's website for references.
Rabbit polyclonal anti-H3, abcam, ab1791, human and mouse, RRID:AB_302613, https://www.abcam.com/products/primary-antibodies/histone-h3-antibody-nuclear-marker-and-chip-grade-ab1791.html
Rabbit monoclonal anti-H3K9ac, abcam, ab32129, human, RRID: AB_732920, https://www.abcam.com/products/primary-antibodies/histone-h3-acetyl-k9-antibody-y28-chip-grade-ab32129.html
Rabbit monoclonal anti-H3K14ac, abcam, ab52946, human, RRID: AB_880442, https://www.abcam.com/products/primary-antibodies/histone-h3-acetyl-k14-antibody-ep964y-chip-grade-ab52946.html
Rabbit monoclonal anti-H3K27ac, abcam, ab177178, human and mouse, RRID: AB_2828007, https://www.abcam.com/products/primary-antibodies/histone-h3-acetyl-k27-antibody-ep16602-chip-grade-ab177178.html
Rabbit polyclonal anti-H3K27ac, abcam, ab4729, human, RRID: AB_2118291, https://www.abcam.com/products/primary-antibodies/histone-h3-acetyl-k27-antibody-chip-grade-ab4729.html
Rabbit polyclonal anti-H3K4me1, abcam, ab8895, human, RRID: AB_306847, https://www.abcam.com/products/primary-antibodies/histone-h3-mono-methyl-k4-antibody-chip-grade-ab8895.html
Rabbit polyclonal anti-H3K4me3, abcam, ab8580, human, RRID: AB_306649, https://www.abcam.com/products/primary-antibodies/histone-h3-tri-methyl-k4-antibody-chip-grade-ab8580.html
Rabbit polyclonal anti-H3K9me3, abcam, ab8898, human, RRID: AB_306848, https://www.abcam.com/products/primary-antibodies/

histone-h3-tri-methyl-k9-antibody-chip-grade-ab8898.html
Rabbit polyclonal anti-H3K36me3, abcam, ab9050, human, RRID: AB_306966, https://www.abcam.com/products/primary-antibodies/histone-h3-tri-methyl-k36-antibody-chip-grade-ab9050.html
Mouse monoclonal anti-H3K9ac, ActiveMotif, 61251, human, RRID: AB_2793569, https://www.activemotif.com/catalog/details/61251
Rabbit polyclonal anti-H3K18ac, ActiveMotif, 39755, human and mouse, RRID: AB_2714186, https://www.activemotif.com/catalog/details/39755/histone-h3-acetyl-lys18-antibody-pab-3
Rabbit polyclonal anti-H3K27me3, Millipore, 07-449, human, RRID: AB_310624, https://www.sigmaaldrich.com/US/en/product/mm/07449
Rabbit monoclonal anti-p300, Cell Signaling Technology, 54062S, human and mouse, RRID: AB_2799450, https://www.cellsignal.com/products/primary-antibodies/p300-d2x6n-rabbit-mab/54062?_requestid=31665
Rabbit monoclonal anti-CBP, Cell Signaling Technology, 7389S, human, RRID: AB_2616020, https://www.cellsignal.com/products/primary-antibodies/cbp-d6c5-rabbit-mab/7389
Rabbit monoclonal anti-HA-Tag, Cell Signaling Technology, 3724S, human, RRID: AB_1549585, https://www.cellsignal.com/products/primary-antibodies/ha-tag-c29f4-rabbit-mab/3724
Rabbit polyclonal anti-Acetyl-CBP (Lys1535)/p300 (Lys1499), Cell Signaling Technology, 4771S, human, RRID: AB_2262406, https://www.cellsignal.com/products/primary-antibodies/acetyl-cbp-lys1535-p300-lys1499-antibody/4771
Mouse monoclonal anti-beta-Actin, Sigma, A1978, human and mouse, RRID: AB_476692, https://www.sigmaaldrich.com/US/en/product/sigma/a1978
Mouse monoclonal anti-vinculin, Santa Cruz, sc-25336, human, RRID: AB_628438, https://www.scbt.com/p/vinculin-antibody-h-10
Rabbit polyclonal anti-GST , Santa Cruz, sc-459, human, RRID: AB_631586, https://www.scbt.com/p/gst-antibody-z-5?requestFrom=search
Mouse monoclonal anti-His tag , ZSGB-Bio, TA-02, human, RRID: AB_2801388, http://www.zsbio.com/product/TA-02
In addiction, p300 and CBP antibodies were validated by lack of signal in our HAT sgRNA samples. Acetyl-CBP (Lys1535)/p300 (Lys1499) antibody was validated by lost signal in our D1399A GST-p300 recombinant protein. The specificities of acetyl-histone antibodies were validated by our PTM peptide array.

Eukaryotic cell lines

Policy information about cell lines and Sex and Gender in Research

Cell line source(s)	DLD-1, H1299, A549, and HEK293T were purchased directly from ATCC. The ATCC cell lines OVCAR-3 and OVCAR-8 were gifted from Jose M. Teixeira at Michigan State University. OVCAR-5 (Sigma) was gifted from Xiongbin Lu at Indiana University School of Medicine. The ATCC cell lines ES-2 and SKOV3 were gifted from Hui Shen at Van Andel Institute.
Authentication	Cell lines from ATCC were authenticated by STR profiling by the vendor. All other cell lines were authenticated at MDACC and MSU by STR profiling.
Mycoplasma contamination	Cells were examined every half year for possible mycoplasma contamination using PCR and they were negative.
Commonly misidentified lines (See ICLAC register)	No commonly misidentified cell lines were used.

Animals and other research organisms

Policy information about studies involving animals; ARRIVE guidelines recommended for reporting animal research, and Sex and Gender in Research

Laboratory animals	5-weeks old female homozygous athymic nude mice (J:NU, 007850) were purchased from The Jackson Laboratory and housed at Van Andel Institute vivarium center. All mice were used at 6-8 weeks old.
Wild animals	No involved.
Reporting on sex	We didn't consider the influence of sex in study design. Female mice were used for all animal assay as reported in the literature studies.
Field-collected samples	No involved.
Ethics oversight	All animal procedures were approved by the Institutional Animal Care and Use Committee (IACUC) at Van Andel Institute.

Note that full information on the approval of the study protocol must also be provided in the manuscript.

Plants

Seed stocks	*Report on the source of all seed stocks or other plant material used. If applicable, state the seed stock centre and catalogue number. If plant specimens were collected from the field, describe the collection location, date and sampling procedures.*
Novel plant genotypes	*Describe the methods by which all novel plant genotypes were produced. This includes those generated by transgenic approaches, gene editing, chemical/radiation-based mutagenesis and hybridization. For transgenic lines, describe the transformation method, the number of independent lines analyzed and the generation upon which experiments were performed. For gene-edited lines, describe the editor used, the endogenous sequence targeted for editing, the targeting guide RNA sequence (if applicable) and how the editor was applied.*

Authentication

Describe any authentication procedures for each seed stock used or novel genotype generated. Describe any experiments used to assess the effect of a mutation and, where applicable, how potential secondary effects (e.g. second site T-DNA insertions, mosiacism, off-target gene editing) were examined.

ChIP-seq

Data deposition

☒ Confirm that both raw and final processed data have been deposited in a public database such as GEO.

☒ Confirm that you have deposited or provided access to graph files (e.g. BED files) for the called peaks.

Data access links
May remain private before publication.

To review GEO accession GSE193648:
Go to https://www.ncbi.nlm.nih.gov/geo/query/acc.cgi?acc=GSE193648

Files in database submission

GSM5815594 OVCAR3 GFP sg1 rep1 H3K27ac
GSM5815595 OVCAR3 GFP sg1 rep2 H3K27ac
GSM5815596 OVCAR3 GFP sg1 rep3 H3K27ac
GSM5815597 OVCAR3 p300 TAZ2 sg1 rep1 H3K27ac
GSM5815598 OVCAR3 p300 TAZ2 sg1 rep2 H3K27ac
GSM5815599 OVCAR3 p300 TAZ2 sg1 rep3 H3K27ac
GSM5815600 OVCAR3 p300 TAZ2 sg2 rep1 H3K27ac
GSM5815601 OVCAR3 p300 TAZ2 sg2 rep2 H3K27ac
GSM5815602 OVCAR3 p300 TAZ2 sg2 rep3 H3K27ac
GSM5815603 OVCAR3 p300 HAT sg1 rep1 H3K27ac
GSM5815604 OVCAR3 p300 HAT sg1 rep2 H3K27ac
GSM5815605 OVCAR3 p300 HAT sg1 rep3 H3K27ac
GSM5815606 OVCAR3 input
GSM5815607 DLD-1 GFP sg1 H3K27ac
GSM5815608 DLD-1 p300 TAZ2 sg1 H3K27ac
GSM5815609 DLD-1 p300 TAZ2 sg2 H3K27ac
GSM5815610 DLD-1 p300 HAT sg1 H3K27ac
GSM5815611 DLD-1 CBP TAZ2 sg1 H3K27ac
GSM5815612 DLD-1 CBP TAZ2 sg2 H3K27ac
GSM5815613 DLD-1 CBP HAT sg1 H3K27ac
GSM5815614 DLD-1 input
GSM5815594 OVCAR3 GFP sg1 rep1 H3K27ac.bigwig
GSM5815595 OVCAR3 GFP sg1 rep2 H3K27ac.bigwig
GSM5815596 OVCAR3 GFP sg1 rep3 H3K27ac.bigwig
GSM5815597 OVCAR3 p300 TAZ2 sg1 rep1 H3K27ac.bigwig
GSM5815598 OVCAR3 p300 TAZ2 sg1 rep2 H3K27ac.bigwig
GSM5815599 OVCAR3 p300 TAZ2 sg1 rep3 H3K27ac.bigwig
GSM5815600 OVCAR3 p300 TAZ2 sg2 rep1 H3K27ac.bigwig
GSM5815601 OVCAR3 p300 TAZ2 sg2 rep2 H3K27ac.bigwig
GSM5815602 OVCAR3 p300 TAZ2 sg2 rep3 H3K27ac.bigwig
GSM5815603 OVCAR3 p300 HAT sg1 rep1 H3K27ac.bigwig
GSM5815604 OVCAR3 p300 HAT sg1 rep2 H3K27ac.bigwig
GSM5815605 OVCAR3 p300 HAT sg1 rep3 H3K27ac.bigwig
GSM5815606 OVCAR3 GFP sg1 input.bigwig
GSM5815607 DLD1 p300 GFP sg1 H3K27ac.bigwig
GSM5815608 DLD1 p300 TAZ2 sg1 H3K27ac.bigwig
GSM5815609 DLD1 p300 TAZ2 sg2 H3K27ac.bigwig
GSM5815610 DLD1 p300 HAT sg1 H3K27ac.bigwig
GSM5815611 DLD1 CBP TAZ2 sg1 H3K27ac.bigwig
GSM5815612 DLD1 CBP TAZ2 sg2 H3K27ac.bigwig
GSM5815613 DLD1 CBP HAT sg1 H3K27ac.bigwig
GSM5815614 DLD1 p300 GFP sg1 input.bigwig
GSM5885686 DLD1 GFP sg1 H3K4me1.bigwig
GSM5885687 DLD1 p300 TAZ2 sg1 H3K4me1.bigwig
GSM5885688 DLD1 p300 TAZ2 sg2 H3K4me1.bigwig
GSM5885689 DLD1 p300 HAT sg1 H3K4me1.bigwig
GSM5885690 DLD1 CBP TAZ2 sg1 H3K4me1.bigwig
GSM5885691 DLD1 CBP TAZ2 sg2 H3K4me1.bigwig
GSM5885692 DLD1 CBP HAT sg1 H3K4me1.bigwig

Genome browser session
(e.g. UCSC)

Bigwig files for genome browser were uploaded in GSE193648.

Methodology

Replicates

Three biological replicates were done for OVCAR-3 H3K27ac ChIP-seq, one biological replicate in DLD-1 H3K27ac ChIP-seq.

Sequencing depth

ChIP-seq data are 100 bp paired-end reads.
sample total_reads mapped_reads

	OVCAR3_GFP_sg1_rep1_H3K27ac 101812433 93402726<br>OVCAR3_GFP_sg1_rep2_H3K27ac 61280271 56855835<br>OVCAR3_GFP_sg1_rep3_H3K27ac 53036272 48989604<br>OVCAR3_p300_TAZ2_sg1_rep1_H3K27ac 76973974 72170798<br>OVCAR3_p300_TAZ2_sg1_rep2_H3K27ac 71442033 66276774<br>OVCAR3_p300_TAZ2_sg1_rep3_H3K27ac 54061918 50472207<br>OVCAR3_p300_TAZ2_sg2_rep1_H3K27ac 80549405 75063991<br>OVCAR3_p300_TAZ2_sg2_rep2_H3K27ac 72926218 68339159<br>OVCAR3_p300_TAZ2_sg2_rep3_H3K27ac 52446864 49383967<br>OVCAR3_p300_HAT_sg1_rep1_H3K27ac 56529296 51362518<br>OVCAR3_p300_HAT_sg1_rep2_H3K27ac 50573516 46846248<br>OVCAR3_p300_HAT_sg1_rep3_H3K27ac 51457369 47222428<br>OVCAR3_GFP_sg1_rep1_input 91304622 81452853<br>DLD1_p300_GFP_sg1_H3K27ac 54693108 52499914<br>DLD1_p300_TAZ2_sg1_H3K27ac 54343013 52239938<br>DLD1_p300_TAZ2_sg2_H3K27ac 53981622 51719792<br>DLD1_p300_HAT_sg1_H3K27ac 55674960 53286504<br>DLD1_CBP_TAZ2_sg1_H3K27ac 62336152 59774136<br>DLD1_CBP_TAZ2_sg2_H3K27ac 57287049 54955466<br>DLD1_CBP_HAT_sg1_H3K27ac 57630735 55285164
Antibodies	anti-H3K27ac, Abcam, ab177178, RRID: AB_2828007.
Peak calling parameters	Peaks were called with command macs2 callpeak --broad -g hs -q 0.05 --broad-cutoff 0.1.<br>All H3K27ac peaks were use the input files in the same cell line as control.<br>Genome index is generated from GRCh38.primary_assembly.genome.fa and gencode.v29.annotation.gtf by HISAT2 (v2.1.0).
Data quality	FASTQC 0.11.8 is run to check the sequencing quality.
Software	HISAT2 (v2.1.0), MACS2 (v2.1.2), Homer (v4.11), bedtools (v2.27.1), Danpos2 (v2.2.2), and R (v3.5.1) are used for ChIP-seq analysis.
